# Machine learning models for prediction of (Pro)cathepsin–glycosaminoglycan binding free energies based on molecular structure

**DOI:** 10.1016/j.csbj.2025.11.059

**Published:** 2025-12-08

**Authors:** Krzysztof K. Bojarski, Patrick K. Quoika, Martin Zacharias

**Affiliations:** a Department of Physical Chemistry, Gdansk University of Technology, Narutowicza 11/12, Gdansk, Poland; b Center for Functional Protein Assemblies, Technical University of Munich, Ernst-Otto-Fischer-Straße 8, Garching, Germany

**Keywords:** Glycosaminoglycans (GAGs), Molecular dynamics (MD), Machine learning (ML), Binding energy calculation (MM-GBSA), Neural networks (NN), Quantitative structure-activity relationship (QSAR)

## Abstract

Cathepsins are papain-like proteolytic enzymes localized in lysosomes and the extracellular matrix, where they participate in diverse physiological and pathological processes. They are synthesized as inactive precursors—procathepsins—containing a propeptide domain that blocks access to the active site. The activity of (pro)cathepsins can be modulated by glycosaminoglycans (GAGs), which are negatively charged, sulfated polysaccharides. This study aimed to develop machine learning (ML) models to predict MM-GBSA binding free energies in (pro)cathepsin–GAG complexes. Molecular dynamics simulations were performed using the ff14SB/GLYCAM06j force field for six (pro)cathepsins and six GAGs, representing four periodic states and six binding poses. Structural and energetic descriptors derived from these simulations were used as input features for eight ML algorithms: ElasticNet, Linear Regression, LinearSVR (with RBFSampler), LightGBM, Histogram Gradient Boosting, Fully Connected Neural Network (FCNN), and Random Forest. The FCNN yielded the most accurate predictions ($R^{2}$ = 0.7124 ± 0.0089; MAE = 5.2033 ± 0.0876 kcal/mol), with GradientBoost-based models performing comparably. Optimal FCNN performance was achieved with a minimal architecture (no hidden layers, dropout rate 0.01, ReLU activation). Incorporating Linear Interaction Energy (LIE) components significantly improved prediction accuracy, and approximately 17,000 data points were sufficient for stable model performance. Overall, this study provides a proof of concept for using ML to estimate binding free energies in protein–GAG systems and establishes a foundation for generalizable, structure-based predictors applicable to a broad range of biomolecular complexes. Beyond predictive accuracy, this approach enables rapid screening of MMGBSA interactions, facilitating the identification of favorable binding regions and accelerating structure-guided design efforts.

## Graphical Abstract

**Figure:**
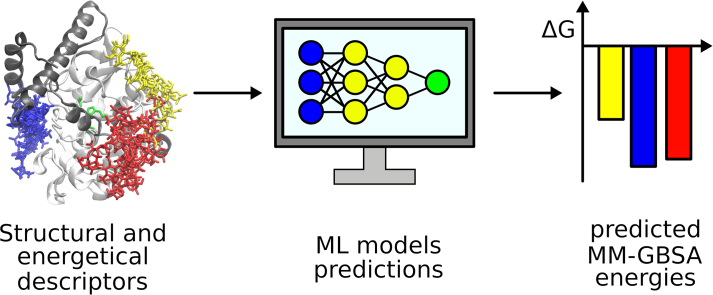

## 1 Introduction

Glycosaminoglycans (GAGs), negatively charged, unbranched and linear polysaccharides [1] are composed of amino sugar/uronic acid disaccharide units. With high heterogeneity and varying chain lengths from 10 to 30 000 disaccharide units [2], [3], [4], [5], [6] they can be classified into chondroitin sulfate (CS), dermatan sulfate (DS), heparin (HP), heparan sulfate (HS), keratan sulfate (KS) and hyaluronic acid (HA), the only GAG present in free state. By binding to their protein targets, including cathepsins [7], procathepsins [8], growth factors and others, they regulate their enzymatic activity. This aspect is crucial since dysregulation of these enzymes may lead to severe diseases, including cancer [9], autoimmune disorders [10], Alzheimer’s disease [11], Parkinson’s disease [12], arthritis [13], and mucopolysaccharidoses [14], [15]. Therefore, in order to effectively treat these disorders and propose novel therapies targeting diseases associated with dysregulated GAG-protein interactions, it is essential to understand these biological processes at atomic level. In that regard, computational approaches show promising results, taking into consideration high resolution of these approaches.

Despite their linear structure, modeling of GAG-containing systems remains a substantial challenge. This originates from the high conformational freedom of these molecules [16], since GAGs monosaccharide rings and glycosidic linkages may adopt different conformations [17], which affects strength of protein-GAG interactions [18]. Since GAGs are strongly negatively charged, most of their interactions are electrostatic-driven which confers an additional challenge in GAG-protein identification [19]. Furthermore, ions [20] and solvent molecules also play crucial role in these interactions [21]. GAGs of the same class (for example chondroitin sulfate) may have different distributions of sulfate groups and yet bind with comparable affinity to their protein targets [22]. These carbohydrates can also bind at different binding poses with comparable energies, phenomenon known as multipose binding [23], [24], with a special case where same GAGs bind at exactly same region with indistinguishable energies but antiparallel conformations [25]. Despite recent developments, there is a scarcity of GAG-dedicated approaches [26], and with a small number of experimentally-available protein-GAG structures (according to GAG-DB database [27]) and thermodynamic data, application of Machine Learning (ML) algorithms remains a substantial challenge [28].

Computational protocols to investigate protein-GAG interactions were designed while taking into consideration these challenges. To characterize GAG-containing systems, it is necessary to predict of GAG binding poses in order to obtain protein-GAG complex structures, perform molecular dynamics simulations (MD) to characterize structural evolution of these systems and calculate binding free energies to assess their stability. Electrostatic potential maps are calculated with the Poison-Boltzmann Surface Area (PBSA) approach, as implemented in AmberTools [29]. Molecular docking is performed using Autodock3 [30] with parameters dedicated to GAG-containing systems [31]. Most commonly used force fields to perform classic MD simulations for protein-GAG systems are ff14SB/GLYCAM06j [29], [32], [33] and CHARMM36m [34], [35] but coarse-grained approaches were also proposed to describe these systems [36]. Furthermore, methods with increased sampling such as steered MD [37], [38] and Repulsive Scaling Replica Exchange MD (RS-REMD) [39] are also employed to investigate conformational space more thoroughly.

Binding affinities in protein–GAG complexes are most commonly estimated using the Molecular Mechanics Poisson–Boltzmann Surface Area (MM-PBSA) method, and for GAG-containing sytems is frequently applied in conjunction with the Generalized Born approximation (MM-GBSA). In this method a notable limitation is its precision. For GAG-containing systems, standard deviations from energies averaged over the simulations may reach up to 20 kcal/mol [40], which further highlights the challenge of applying this method for estimation of absolute binding free energy. In MM-GBSA calculations, the entropic contribution is typically estimated using normal-mode analysis, which relies on additional approximations and structural truncation, often leading to significant statistical uncertainty and limited reliability of the resulting binding free energy estimates [41]. Therefore, this method is effectively employed to compare the relative binding affinities of structurally similar complexes. In this context, MM-GBSA energy values are commonly used to score structures obtained from RS-REMD simulations. In recent studies using the ff14SB/GLYCAM06j force fields, it was demonstrated that the top 10 structures (selected based on MM-GBSA scores) exhibited high structural similarity to experimentally determined complexes [42], [43]. Moreover, in another study, comparative MM-GBSA analysis of different cathepsin–GAG complexes enabled the explanation of observed differences in enzymatic activity in the presence of GAGs [44]. The MD/MM-GBSA approach also allowed to identification and characterization of binding sites in cyclic peptide-GAG complexes [45]. These findings support the view that, despite known limitations in precision, MM-GBSA remains a valuable method for assessing the stability and interaction profiles of protein–GAG complexes.

Alternative strategies for estimating binding affinities in receptor–ligand systems are ML algorithms or empirical scoring functions in combination with molecular docking. One such example is DockTScore, which integrates physics-based descriptors with ML techniques and has shown improved correlation with experimental binding affinities compared to traditional empirical scoring functions [46]. It also demonstrated enhanced performance in ranking active compounds during virtual screening. Similarly, Zhang et al. [47] developed the Artificial Intelligence-based Scoring Function Platform (ASFP), which achieved strong predictive accuracy on benchmark datasets—reporting an average AUC of 0.973 across 32 targets from the DUD-E set and a Pearson correlation of 0.81 on the PDBbind 2016 core set. In the case of protein-GAG complexes, the performance of various ML models—including Fully Connected Neural Networks, LightGBM, Linear Regression, Rand. Forest  and Support Vector Regression—was evaluated for the prediction of Root Mean Square atom type deviation (RMSatd) based on energy terms and structural descriptors [48]. The application of ML models resulted in improved correlation between predicted and reference RMSatd values compared to predictions based solely on MM-GBSA energies. Moreover, for nearly all tested algorithms, the selection of the top 10 most favorable binding poses showed greater structural similarity to the experimental complex. Among the models evaluated, Rand. Forest consistently provided the most accurate predictions. These studies illustrate the potential of ML-driven scoring functions to improve affinity prediction and support structure-based drug discovery.

**Fig. 1:**
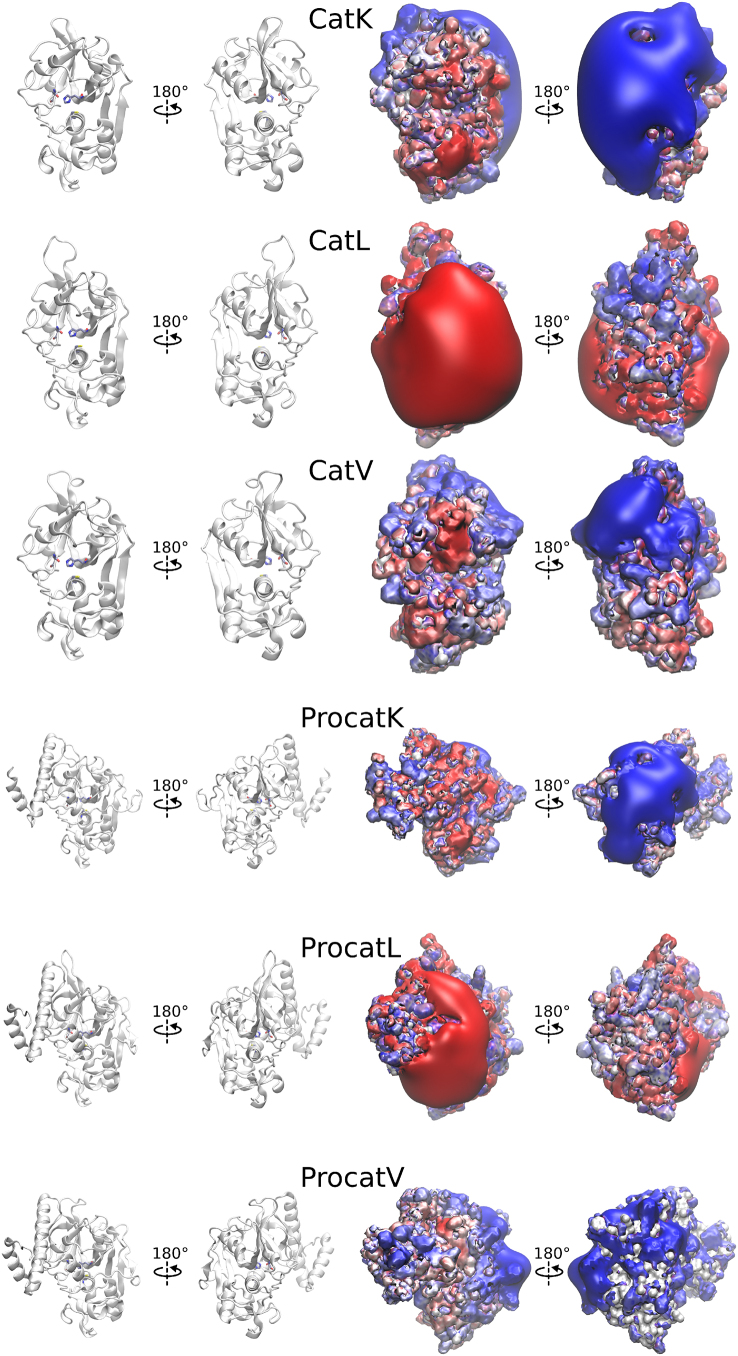
Structures of (Pro)cathepsins K, L and V, whose complexes with GAGs were characterised in this study (PDB IDs are provided in Section 2.1) along with their calculated electrostatic potential isosurfaces in surface representation (red, −3 kcal/(mol⋅e); blue, +3 kcal/(mol⋅e), respectively). Electrostatic potential isosurfaces were calculated with “pbsa” module of AMBER [29] and visualised with VMD [49]. (For interpretation of the references to colour in this figure legend, the reader is referred to the web version of this article.)

While previous studies have applied ML models to MM-GBSA predictions or protein–GAG systems separately, a systematic evaluation and direct comparison of multiple ML algorithms on a diverse set of protein–GAG complexes has not yet been reported. In this study, we aimed to investigate the performance of different ML models–specifically, Elastic Net, Fully Connected Neural Network (FCNN), Hist. Grad. Boost, LightGBM, Linear Regression, Linear SVR (including a version with RBF sampler as separate model) and Rand. Forest–to predict MM-GBSA binding free energies based on structural descriptors. The ML models were trained and tested on complexes of six (pro)cathepsins (Fig. 1) with six types of GAGs of four lengths and at different binding poses. Despite their high structural similarity, each (pro)cathepsin exhibits a distinct electrostatic potential isosurface. The protein data set includes (pro)cathepsin K, which displays large, well-defined positively charged surface patches; (pro)cathepsin L, which in contrast to the former, features extensive negatively charged regions; and (pro)cathepsin V, which presents an intermediate electrostatic profile between the two. To ensure robustness and diversity of the training data, each complex was simulated using all-atom MD in explicit solvent, followed by MM-GBSA binding free energy calculation. From the resulting MD trajectories, a set of structural and energetic descriptors was extracted and used as input features for ML models development for which, correlation analysis was performed. In addition to comparing general performance of different algorithms, we also evaluated the influence of different input features on model accuracy, in particular, Linear Interaction Energy (LIE) components. Furthermore, we optimized the hyperparameters of the neural network models, including network architecture, dropout rate and activation function. In addition, we demonstrated how the best-performing ML model can be applied for fast evaluation of MMGBSA binding interactions, highlighting its potential for future large-scale screening studies. The presented workflow aims to demonstrate the feasibility of ML-based approaches to estimate binding energetics in protein–GAG systems and provide a foundation for further methodological improvements tailored to GAG-containing complexes.

## 2 Materials and methods

### 2.1 Systems preparation

In this study, six (pro)cathepsins complexes with six different types of GAGs were analyzed (Table 1). The structures of the protein targets were obtained from the PDB as well as from the AlphaFold predictions. GAG structures were built using the tleap module from the AMBER20 suite [29], based on monomeric building blocks from the sulfated GAG unit libraries [33]. Atomic charges were assigned according to the GLYCAM06j force field [32], with sulfate group parameters taken from the literature [50]. Oligosaccharides of varying lengths — dimers (dp2), tetramers (dp4), hexamers (dp6), and octamers (dp8) — were modeled for each GAG. At the beginning of the simulations, each GAG was initially positioned on the protein surface in six distinct locations: front, back, top, bottom, left, and right, relative to the orientation defined by the respective experimental or predicted protein structure.

**Table 1:** PDB and AlphaFold IDs for the structures of the (pro)cathepsins along with the modeled dimeric unit composition of GAGs investigated in this study.

(Pro)cathepsin type	Cathepsin (Cat)	Procathepsin (ProCat)
K	3C9E [51]	1BY8 [52]
L	2XU3 [53]	1CS8 [54]
V	1FH0 [55]	AF-O60911-F1

GAG name	GAG abbrev.	Dimeric unit

chondroitin 4-sulfate	C4-S	GalNAc(4S)-GlcA
chondroitin 6-sulfate	C6-S	GalNAc(6S)-GlcA
dermatan sulfate	DS	GalNAc(6S)-IdoA
hyaluronic acid	HA	GlcNAc-GlcA
heparin	HP	GlcNS(6S)-IdoA(2S)
heparan sulfate	HS	GlcNAc-IdoA

### 2.2 Molecular dynamics

Protein-GAG complexes were first solvated in a TIP3P [56] octahedral periodic box with a layer of water molecules of 8 Å from the border of the periodic box to the solute and neutralized with counterions (Na+ or Cl- depending on the overall charge of a system). For protein and GAG parts of the system ff14SB [57] and GLYCAM06j [32] force field parameters were used, respectively. Energy minimization was carried out in two steps: first, 0.5 × 10^3^ steepest descent cycles and 10^3^ conjugate gradient cycles with harmonic force restraints of 100 kcal/(mol⋅Å^2^) on solute atoms and then, 3 × 10^3^ steepest descent cycles and 3 × 10^3^ conjugate gradient cycles without restraints. Afterward, the system was heated up to 300 K for 10 ps with harmonic force restraints of 100 kcal/(mol⋅Å^2^) on solute atoms. The systems were equilibrated for 5 ns at 300 K and 1 atm in the isothermal-isobaric (NPT) ensemble using Langevin dynamics with a collision frequency of 1.0 ps^-1^. During this stage, harmonic distance restraints (10 kcal/(mol⋅Å^2^)) were applied between the C1 atom of each monosaccharide unit and the C${\;}_{\alpha }$ atom of the protein residue closest to the center of mass. The target distance for each restraint was calculated individually for each complex in order to guide the glycosaminoglycan (GAG) chain toward the protein surface.

Finally, a 25 ns productive MD run was carried out in an NPT ensemble. The SHAKE algorithm, 2 fs time integration step, an 8 Å cutoff for non-bonded interactions and the particle mesh Ewald method were used. The structures were written every 25 ps, which produced 10^3^ frames in total per simulation used for further analysis. The resulting dataset used for model training consisted of 841,132 data points, with frames in which the GAG was dissociated from the (pro)cathepsin excluded.

### 2.3 Binding free energy analysis

Energetic postprocessing of the trajectories for all protein–GAG complexes was carried out using the molecular mechanics generalized Born surface area (MM-GBSA) approach, employing a continuum solvent model with default surface area and Born radii parameters as implemented in the igb = 2 model of AMBER20 [29], [58]. All frames from the MD simulations were included in the subsequent analyses.

### 2.4 Calculation of the structural descriptors for machine learning models training

To derive structure-based descriptors suitable for machine learning (ML) model training, a set of custom Python scripts was developed using the MDAnalysis library [59] as well as the cpptraj module of AMBER20 [29]. These tools were applied to process molecular dynamics (MD) trajectories of protein–glycosaminoglycan (GAG) complexes and extract quantitative structural and physicochemical features characterizing the spatial arrangement and interaction profile of each complex. As no interactions crossing periodic boundaries were observed, they were not included in these analyses.

Descriptors related to electrostatic interactions included the total formal charges of the protein and GAG, computed by summing atomic charges defined in the ff14SB and GLYCAM06j force fields. Additionally, the numbers of positively charged (Lys, Arg, His, Hie) and negatively charged (Asp, Glu) protein residues located within 5 Å of the GAG were calculated. From these counts, the local net charge within 5 Å and 10 Å of the GAG was derived by subtracting the number of nearby negative residues from positive residues.

To capture geometric and conformational features of the complexes, the end-to-end distance (EED) and radius of gyration (RadGyr) of the GAG were computed. The center-of-mass (COM) distance between the GAG and the protein, as well as the GAG orientation angle (defined as the angle between the principal axes of the protein and the ligand), were also included to reflect the spatial positioning and alignment of the GAG relative to the protein surface.

Descriptors reflecting the polarity of the local interaction environment were computed by classifying protein residues into hydrophobic (Ala, Val, Leu, Ile, Phe, Trp, Met, Tyr) and hydrophilic (Ser, Thr, Asn, Gln, Asp, Glu, Lys, Arg, His, Hie) groups. For both 5 Å and 10 Å radii around the GAG, the number of nearby hydrophobic and hydrophilic residues was calculated, as well as a polarity index defined as the difference between hydrophilic and hydrophobic counts.

Additional interaction-based descriptors were calculated using the cpptraj module. These included the number of (pro)cathepsin–GAG contacts (via the nativecontacts command, with contact distance thresholds of 3.5 Å for short and 5.0 Å for medium-range contacts), the number of hydrogen bonds (using the hbond command, with separate analysis of GAG as donor and acceptor; hydrogen bonds were defined by a 3.5 Å donor–acceptor distance cutoff and a 135${\;}^{\circ }$ angle cutoff), the solvent-accessible surface area (SASA) of the protein, GAG, and entire complex (via the surf command), and the Linear Interaction Energy (LIE) between protein and GAG (via the lie command, with protein defined as receptor and GAG as ligand).

Together, this set of descriptors captures electrostatic, geometric, and interaction-based features of the protein–GAG interface and was used as input for ML model development.

### 2.5 Development of machine learning models to predict MM-GBSA binding free energies

To predict MM-GBSA binding free energies based on the structural descriptors defined in previous section, a set of eight ML models was trained. Specifically, the methods used are (including abbreviations for usage below):

• Elastic Net Regression (Elastic Net) [60]
• Fully Connected Neural Network (FCNN) [61]
• Histogram-based Gradient Boosting Regressor (Hist. Grad. Boost) [62]
• Linear Regression (Lin. Reg.) [63]
• Light Gradient Boosting Machine (LightGBM) [64]
• Linear Support Vector Regressor (Lin. SVR)
• Random Forest (Rand. Forest) [65]
• Radial Basis Function sampler in combination with Linear SVR (RBFSampler + Lin. SVR)

In the following, we provide the details of these different approaches separately.

All ML models in this study used StandardScaler for feature normalization to zero mean and unit variance. Model performance was evaluated using the coefficient of determination ($R^{2}$), mean squared error (MSE), and mean absolute error (MAE), which were consistently reported for both training and test sets. For all models developed in this study, the dataset was split into 80 % train and 20 % test set with the split being regenerated independently each time a new model was trained. Furthermore, for each ML algorithm (considering all descriptors), we trained 10 independent models.

Furthermore, the accuracy of all ML models was evaluated on an independent validation set, composed of cathepsin S-GAG hexamer complexes. These complexes were obtained from our previous study [44] and were not included in any stage of model training or initial testing. Thus, the validation dataset, of 37,981 data points, offers a rigorous test of the models’ generalizability to unseen data from the same protein–GAG class but with a different cathepsin subtype. Such external validation is crucial to demonstrate the robustness and transferability of the models in predicting MM-GBSA binding energies across related but distinct molecular complexes.

To account for potential multicollinearity and enable feature selection while preserving generalizability, we employed the ElasticNet regression model. This method linearly combines L1 (Lasso) and L2 (Ridge) regularization terms, controlled via the l1_ratio parameter. The model was implemented using the ElasticNet class from scikit-learn, with regularization strength α = 1.0 and equal weighting of L1 and L2 penalties (l1_ratio = 0.5).

A classical linear regression model was used as a baseline for predicting MM-GBSA binding energies. The model maps molecular descriptors to the target energy using a simple weighted sum of input features with an added intercept term. No regularization or higher-order terms were introduced, ensuring a strictly linear relationship between inputs and the predicted output. The model was implemented using the LinearRegression class from scikit-learn.

To assess the predictive performance of a tree-based ensemble method, we employed a Light Gradient Boosting Machine (LightGBM) regressor. This model is known for its efficiency and scalability, particularly with structured tabular data. For this dataset, we fine-tuned the following hyperparameters: the number of boosting rounds (n_estimators = 100, **200**), the maximum tree depth (max_depth = 4, 6, **8**), the learning rate (learning_rate = 0.05, **0.1**), and the subsampling ratios (**0.8**) for both rows (subsample) and columns (colsample_bytree). The parameters corresponding to the best-performing model are indicated in bold. The model was trained on the scaled training set and evaluated on the held-out test set.

To evaluate an alternative ensemble learning strategy for MM-GBSA energy prediction, we employed the Histogram-based Gradient Boosting Regressor (HistGradientBoostingRegressor) from the scikit-learn library. This algorithm implements a binning-based version of gradient boosting that significantly improves computational efficiency by discretizing continuous features into integer bins. The regressor was fine-tuned by exploring different numbers of boosting iterations (max_iter = 100, **200**), a tree depth (max_depth = 4, **6**), a learning rate (learning_rate = 0.05, **0.1**) and L2 regularization strength (l2_regularization = 0.0, **0.1**). The hyperparameters corresponding to the best-performing model are shown in bold. To quantify prediction uncertainty, we performed 30 rounds of 10-fold cross-validation on the test set using the same model configuration and computed the standard deviation of predictions across runs.

The Fully Connected Neural Network (FCNN) also known as Multilayer Perceptron (MLP) model employed for MM-GBSA energy prediction consisted of a feed-forward architecture with a variable number of hidden layers. Model training was performed in two stages. In the first stage, various architectures were explored, including networks with zero to three hidden layers (24-1, 24–12-1, 24–12-6–1, 24–12-6–3-1), all using ReLU activation. Different dropout rates (0, 0.01, 0.05, 0.3) were tested to identify the optimal regularization strength, with 0.01 yielding the best performance. In the second stage, with dropout fixed at 0.01, multiple activation functions were evaluated, including ReLU, ELU, GELU, LeakyReLU, Sigmoid, and Tanh, across the same set of architectures. The final choice of ReLU activation balanced predictive performance and training stability. The network was trained using the Adam optimizer with a learning rate of 0.001 and L2 weight decay of 10^−4^. Training incorporated early stopping with a patience of 15 epochs monitored on the validation loss, and learning rate reduction upon plateau to enhance convergence. To estimate predictive uncertainty, Monte Carlo Dropout was implemented by enabling dropout during inference and averaging predictions over 30 stochastic forward passes.

To further benchmark ensemble learning methods for MM-GBSA energy prediction, we applied the Rand. Forest regressor as implemented in the scikit-learn library. Rand. Forest is a bagging-based algorithm that aggregates predictions from multiple decision trees trained on bootstrapped subsets of the data with feature-level randomization, which helps reduce overfitting and improve robustness. The model was fine-tuned with respect to the following hyperparameters: the number of decision trees (n_estimators = 50, 100, **200**), the maximum tree depth (max_depth = 10, 20, **30**, None), the minimum number of samples required to split an internal node (min_samples_split = **2**, 5, 10), the minimum number of samples required to be at a leaf node (min_samples_leaf = **1**, 2, 4), and the number of features considered when looking for the best split (max_features = **‘sqrt’**, ‘log2’, None). The hyperparameters corresponding to the best-performing model are highlighted in bold. This configuration balances model complexity with generalization, allowing the regressor to capture nonlinear dependencies in the input descriptors while maintaining predictive stability.

To evaluate a linear kernel–based regression approach for MM-GBSA energy prediction, we employed the Linear Support Vector Regressor (LinearSVR) from the scikit-learn library. This model seeks to find a linear function that approximates the target values while allowing for a small margin of tolerance (ϵ = 0.1) and controlling model complexity via the regularization parameter (C = 1.0). The maximum number of iterations was set to 10,000 to ensure convergence. LinearSVR was chosen over the standard SVR with non-linear kernels due to the large size of the dataset. Using a full SVR would have been computationally prohibitive, whereas LinearSVR provides an efficient alternative capable of handling large-scale data while still capturing the linear dependencies between descriptors and MM-GBSA binding free energies.

To investigate the potential benefits of capturing non-linear relationships in MM-GBSA energy prediction while maintaining computational efficiency, we employed a pipeline combining an RBFSampler with a Linear Support Vector Regressor (LinearSVR) from the scikit-learn library. The RBFSampler approximates the radial basis function (RBF) kernel by projecting the original feature space into a higher-dimensional space, enabling the linear model to capture non-linear patterns in the data. The LinearSVR component was configured with a regularization parameter C = 1.0, ϵ = 0.1, and a maximum of 10,000 iterations to ensure convergence. This approach was chosen to extend the original LinearSVR model with kernel-based feature transformations, thereby enhancing its ability to model complex dependencies between structural descriptors and MM-GBSA binding free energies without incurring the high computational cost of a full non-linear SVR. The combination of RBFSampler and LinearSVR thus provides a balance between capturing non-linear effects and maintaining scalability for large datasets, offering a practical alternative for energy prediction tasks where standard SVR would be computationally prohibitive.

## 3 Results and discussion

### 3.1 Molecular description of the interaction descriptors relevant to stability of (pro)cathepsin-GAG complexes

**Fig. 2:**
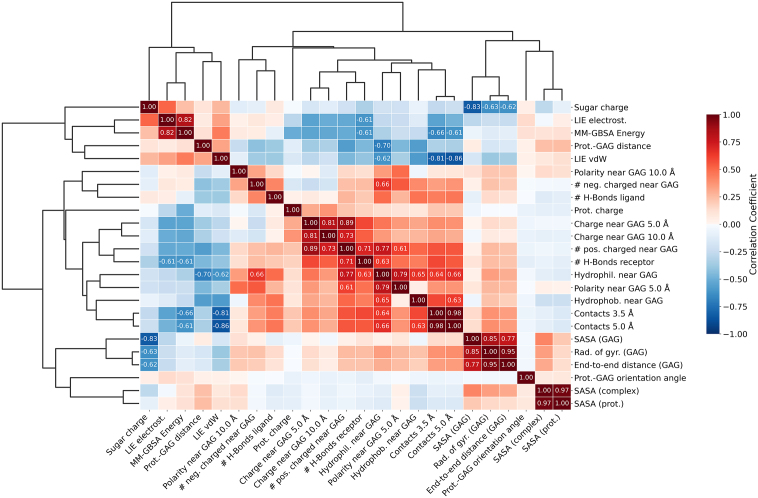
Correlation matrix of features describing (pro)cathepsin–GAG interactions, with hierarchical clustering of features.

In the first step of our machine learning (ML) model development, we aimed to calculate molecular descriptors that could serve as input features and play a crucial role in determining (pro)cathepsin–GAG complex stability. This general approach was successfully applied in our previous work, where ML models were used to predict Root Mean Square atom type deviation (RMSatd) based on MM-GBSA total interaction energies, their individual components, and two structural descriptors (the number of hydrogen bonds between ligand and receptor, and the minimum distance between them) [48]. A similar rationale is applied here, with the key difference being that the goal is now to predict interaction energies. Accordingly, our current feature set primarily includes structural descriptors, complemented by selected energetic terms — specifically, the electrostatic and van der Waals components of the Linear Interaction Energy (LIE) method (Fig. 2). The aim of this descriptor-based analysis is to understand how individual molecular features contribute to (pro)cathepsin–GAG binding energetics.

The molecular descriptors showing the strongest correlation (positive or negative) with MM-GBSA energies include the electrostatic component of the LIE method (0.822), the number of short-range (−0.664) and medium-range (−0.612) contacts, the number of hydrogen bonds in which the (pro)cathepsin acts as a hydrogen bond donor (−0.609), the electrostatic potential at the (pro)cathepsin surface within 5 Å (−0.543) and 10 Å (−0.525) from the GAG molecule, the number of positively charged residues in the (pro)cathepsin (−0.514), and the overall net electrostatic charge of the receptor (−0.507). These results highlight the predominant role of electrostatics in driving (pro)cathepsin–GAG interactions.

Descriptors with moderate (anti)correlation include the van der Waals component of LIE (0.452), the number of hydrophilic (−0.330) and hydrophobic (−0.254) residues in the vicinity of the GAG molecule, the net charge of the GAG (0.236), and the polarity of the (pro)cathepsin surface within 5 Å of the GAG (−0.230). These findings suggest that while polarity contributes to interaction strength, it is likely secondary to electrostatic effects.

Descriptors with weak correlation to MM-GBSA energies and therefore likely to be less influential include the GAG orientation angle (0.125), solvent-accessible surface area (SASA) of the protein (0.124), GAG (−0.058), and the entire complex (0.102), the center-of-mass (COM) distance between (pro)cathepsin and GAG (0.124), GAG radius of gyration (−0.119) and end-to-end distance (−0.108), the number of hydrogen bonds where the GAG acts as a donor (−0.086), the polarity of the protein surface within 10 Å from the GAG (0.067), and the number of negatively charged residues near the GAG (0.062). These results indicate that purely structural or geometrical descriptors likely play a minor role in determining complex stability, at least in comparison to electrostatic interactions.

The obtained correlation heatmap reveals that several descriptors are mutually correlated. To investigate redundancy we performed hierarchical clustering of the correlation matrix, which strongly highlights co-varying descriptor pairs (e.g., SASA (prot.) — SASA (complex); Rad. of gyr. (GAG) — End-to-end distance (GAG); # H-bonds (ligand donor) — # pos. charged near GAG). Such clustering can directly guide feature reduction by selecting one representative from each tightly clustered group, preserving interpretability while removing collinearity. Alternatives for selection strategies could involve: (i) principal component analysis (PCA) to obtain orthogonal low-dimensional representations when interpretability is less critical; (ii) regularized models (L1-penalty/Lasso) or embedded selection in tree-based learners (feature importance in XGBoost) to identify sparse informative subsets; and (iii) permutation-based importance computed with grouped or conditional permutations to avoid confounding by correlated electrostatic descriptors.

In comparison to the correlations obtained for descriptors used during ML model training, their distribution shows noticeable differences in the validation set (Figure S1). The most pronounced variations are observed for the LIE electrostatic component (0.128), SASA of GAG (0.706), sugar charge (–0.520), and the electrostatic potential on the (pro)cathepsin surface within 10 Å of the GAG molecule (0.167). These shifts suggest that the physicochemical landscape of the validation set differs from that of the training data, particularly in terms of electrostatic and solvation-related features. Consequently, the validation dataset serves as a robust test for model generalization and prevents overfitting to the structural characteristics present in the training data.

In summary, our feature correlation analysis underscores the central role of electrostatic interactions in determining the stability of (pro)cathepsin–GAG complexes. Energetic descriptors, particularly electrostatic terms from LIE, alongside features reflecting charged interactions and short- to medium-range contacts, appear most predictive of MM-GBSA energies. In contrast, geometric features and global shape-related properties of the GAG molecule have significantly lower impact. These insights support the hypothesis that electrostatics dominate (pro)cathepsin–GAG recognition and can guide further optimization of ML models by emphasizing the most informative features.

### 3.2 Conformational dynamics of GAGs in molecular dynamics simulations

To assess the extent of conformational diversity sampled by the GAGs during the MD simulations, we evaluated the time evolution of the GAG Root Mean Square Deviation (RMSD) for all investigated complexes, including the three cathepsins and their corresponding procathepsins bound to C4-S (dp8). For each protein–GAG system, six independent MD trajectories were analyzed, each initiated from a distinct binding pose as described in 2.1, 2.2. The RMSD of the GAG relative to its initial conformation in each trajectory was calculated and subsequently averaged to obtain a representative RMSD profile for each complex, along with its associated standard deviation. This analysis was performed to evaluate whether our MD protocol allows the GAGs to explore a sufficiently broad conformational space.

The resulting profiles (Figure S2) reveal that C4-S undergoes substantial conformational rearrangements over the course of the simulations. In the cathepsin complexes, the mean RMSD values gradually increase and reach approximately 20 Å by the end of the trajectories. Even larger conformational excursions are observed in the procathepsin complexes, where the mean RMSD approaches or exceeds 25 Å. The corresponding standard deviations are also considerable, in some cases nearing ∼20 Å (particularly for the (pro)cathepsin L systems), indicating that the individual simulations sample markedly distinct regions of conformational space.

Overall, these results show that—under the present simulation conditions—the GAGs do not remain confined to a single bound conformation. Instead, they explore a wide range of configurational states, alleviating concerns about limited sampling or excessive conformational correlation within the MD dataset. The provided RMSD profiles thus confirm that the MD simulations generate structurally heterogeneous GAG ensembles, supporting the suitability of these data for subsequent machine-learning analyses.

### 3.3 Development of a machine learning models for predicting MM-GBSA energies

Taking into account all molecular descriptors characterized in the previous section, we trained eight ML models to predict MM-GBSA binding free energies. The selected models, including ElasticNet, FCNN, Hist. Grad. Boost, LightGBM, Linear Regression, LinearSVR (including variant with RBFSampler) and Rand. Forest, represent a diverse set of algorithmic approaches, encompassing linear models, decision tree-based ensembles, and neural networks. This variety ensures that the modeling space includes both interpretable, regularized methods and more flexible, non-linear architectures capable of capturing complex feature interactions.

The predictive accuracy of the ML models was evaluated using a validation set comprising cathepsin S–GAG complexes, which were excluded from model training. This strategy enabled a rigorous assessment of the models’ generalization performance to previously unseen protein–ligand systems.

In developing our ML models, we evaluated a range of algorithms to identify the most accurate one on the validation set. For GradientBoost-based approaches and Rand. Forest, we employed GridSearchCV to optimize hyperparameters. In the case of FCNN development, we performed two stages of testing to identify the best-performing configuration: in the first stage, we explored various neural network architectures and dropout rates; in the second stage, we tested selected architectures (with dropout rates chosen from the first stage) alongside different activation functions. Finally, for all investigated models, we evaluated the contribution of energetic descriptors (LIE electrostatics and van der Waals) to the prediction accuracy of MM-GBSA energies. This analysis aimed to determine whether models would still be able to produce accurate binding free energy predictions in the absence of such components.

#### 3.3.1 Comparison of prediction accuracy of different machine learning models

**Fig. 3:**
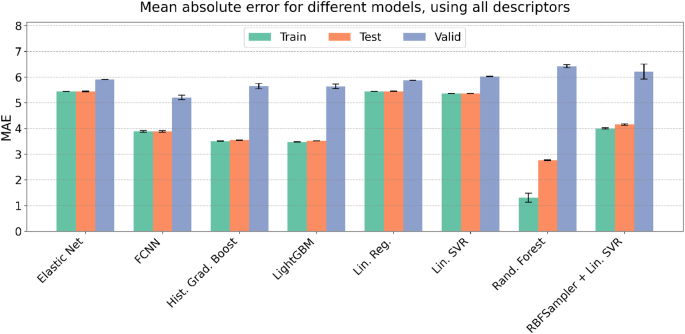
Comparison of the MAE metrics on train, test and validation sets for ML models tested in this study. The values of MAE are given in units of kcal/mol.

To classify the analyzed models in terms of their accuracy in predicting MM-GBSA energies, we primarily considered $R^{2}$, MSE, and MAE metrics obtained for the validation set, as these metrics reflect how well the models generalize to unseen data (Figs. 3, S3, S4). The comparison of different ML approaches demonstrated that the best performance on the validation set was achieved by the FCNN model ($R^{2}$ = 0.7124 ± 0.0089; MAE = 5.2033 ± 0.0876 kcal/mol). This finding suggests that neural network models are particularly suitable for predicting MM-GBSA energies, as they can capture underlying non-linearities in the data. Nevertheless, further optimization and larger datasets may be required to ensure robust generalization.

GradientBoost-based models also showed strong predictive performance. Both HistGradientBoost ($R^{2}$ = 0.6622 ± 0.0127; MAE = 5.6499 ± 0.1042 kcal/mol) and LightGBM ($R^{2}$ = 0.6641 ± 0.0123; MAE = 5.6353 ± 0.0872 kcal/mol) performed competitively. However, the differences between training and validation metrics were more pronounced than for FCNN, indicating a higher susceptibility of GradientBoost-based models to overfitting. The Rand. Forest model, another decision tree–based method, was less accurate than both GradientBoost-based models ($R^{2}$ = 0.5630 ± 0.0082; MAE = 6.4210 ± 0.0631 kcal/mol), and the gap between training and validation performance was even larger, emphasizing the need for careful hyperparameter tuning to mitigate overfitting.

Linear regression–based models were somewhat less accurate than GradientBoost approaches but still provided reasonable predictions given their simplicity. Linear Regression ($R^{2}$ = 0.6351 ± 0.0004; MAE = 5.8779 ± 0.0037 kcal/mol) and LinearSVR ($R^{2}$ = 0.6077 ± 0.0010; MAE = 6.0224 ± 0.0071 kcal/mol) achieved results comparable to GradientBoost-based models. Interestingly, extending LinearSVR with an RBFSampler reduced predictive performance ($R^{2}$ = 0.5923 ± 0.0409; MAE = 6.2120 ± 0.2950 kcal/mol), indicating that kernel approximations do not necessarily improve prediction accuracy in this context. ElasticNet provided predictions comparable to the Linear Regression model ($R^{2}$ = 0.6317 ± 0.0006; MAE = 5.9082 ± 0.0057 kcal/mol).

**Fig. 4:**
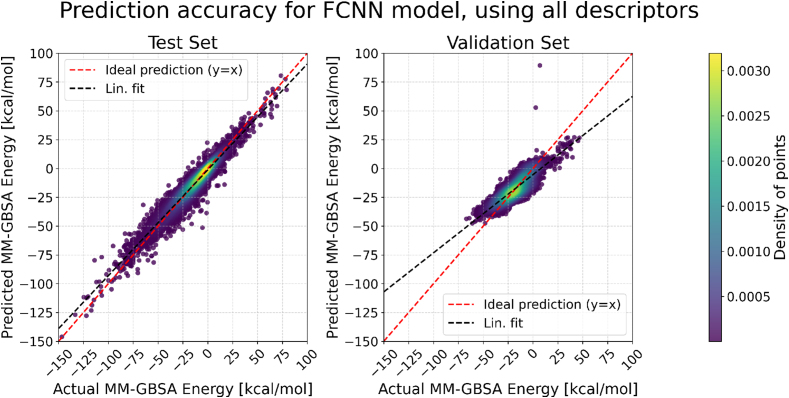
Comparison of actual and predicted MM-GBSA binding free energies for the FCNN model. Scatter plots show the prediction performance on the test set (left), and validation set (right). Color intensity represents the density of points, as estimated using 2D kernel density estimation (KDE). The red dashed line indicates the ideal prediction (y = x). (For interpretation of the references to colour in this figure legend, the reader is referred to the web version of this article.)

A closer inspection of predicted versus actual MM-GBSA energies for the test and validation sets allowed us to identify strengths and weaknesses of each model. For the FCNN model, predictions on the test set closely followed the ideal line. On the validation set, energies of the weakest binders were slightly overestimated, with observed outliers corresponding to complexes with unfavorable LIE_VDW components, indicating areas where the model struggled (Fig. 4). Similar patterns were observed for HistGradientBoost (Figure S5) and LightGBM (Figure S6): predictions for the test set were near ideal, while validation set deviations were slightly larger, with minor overestimation for weakest binders and underestimation for strongest binders. Rand. Forest exhibited strong overfitting: test set predictions were accurate, but the model had difficulty distinguishing the most and least stable complexes on the validation set (Figure S7). Linear Regression showed comparable deviations for both test and validation sets, slightly overestimating energies for weakest binders and underestimating for strongest binders, making discrimination of extreme cases more challenging (Figure S8). LinearSVR exhibited a similar pattern (Figure S9), while adding RBFSampler increased deviations from the ideal line (Figure S10). ElasticNet faced the largest difficulties in distinguishing the most and least stable complexes across both sets (Figure S11).

Overall, these results demonstrate that predicting MM-GBSA binding free energies remains a challenging task. Advanced architectures such as neural networks and gradient-boosting models tend to outperform simple regression-based methods. At the same time, the relatively consistent performance across different algorithms suggests that the dataset contains a robust predictive signal. Further improvements in model design, feature representation, and handling of outliers will likely be necessary to enhance predictive accuracy and generalization further.

#### 3.3.2 Analysis of the feature importance on prediction accuracy

To better understand the impact of specific features on MM-GBSA energy predictions, we analyzed their importance across multiple models. We compared normalized feature weights (LinearSVR), coefficients (ElasticNet), built-in feature importances (Rand. Forest), permutation feature importance (FCNN, HistGradientBoost), SHapley Additive exPlanations (LinearSVR + RBFSampler), and a leave-one-feature-out approach, where the importance of a descriptor was quantified as the MSE difference between a model trained with all descriptors and the same model trained without the descriptor of interest (LightGBM, Linear Regression).

**Fig. 5:**
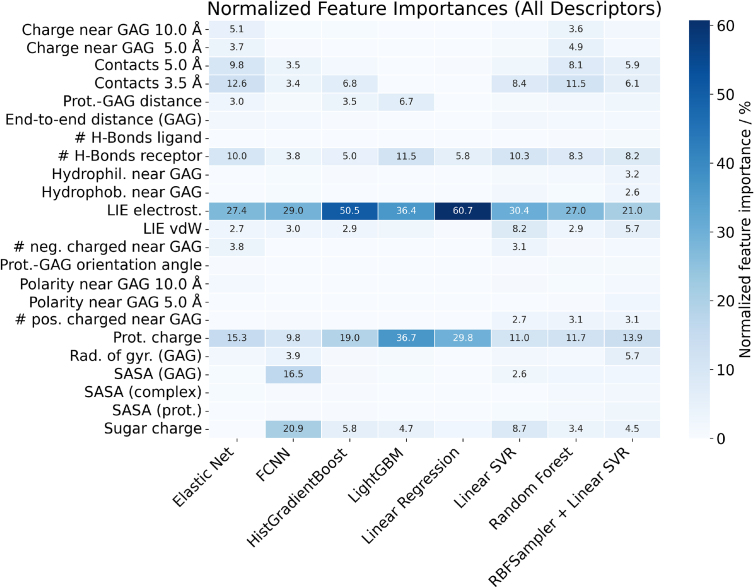
Normalized feature importance for all ML models trained in this study.

The results indicate that for most models, LIE descriptors, especially the electrostatic component (LIE_ELEC), are the dominant contributors (Fig. 5). This effect is most pronounced for Linear Regression, consistent with the previously observed strong correlation between LIE_ELEC and MM-GBSA binding energy. As model complexity increases, additional electrostatics-related descriptors gain importance. For instance, in ElasticNet, HistGradientBoost, LinearSVR (+RBFSampler), and Rand. Forest, the protein charge emerges as highly influential. Other relevant descriptors in these models include the number of hydrogen bonds where the receptor acts as a donor, as well as the number of contacts. In the FCNN model, SASA of the GAG, protein charge, and GAG charge are also significant, indicating that FCNN predictions rely more heavily on electrostatic aspects of (pro)cathepsin–GAG interactions. Among all models, LightGBM provides the most balanced picture of feature importances: while LIE_ELEC remains crucial, other descriptors with comparable relevance include the (pro)cathepsin–GAG distance, GAG SASA, protein charge, and the van der Waals component of LIE.

In summary, these results demonstrate that electrostatic-related descriptors (LIE_ELEC, protein and GAG charge, hydrogen bonds) are the main drivers of MM-GBSA predictions across models. In the absence of explicit LIE terms (which are further discussed in the following section), structural descriptors (contacts, SASA, distance, radius of gyration) increase in importance. It should also be noted that these trends partly reflect the characteristics of the underlying force field, and therefore some force field specificity is expected. Among the tested approaches, LightGBM consistently provides the most balanced distribution of feature importance, whereas Linear Regression and FCNN tend to emphasize electrostatics more strongly.

#### 3.3.3 Importance of linear interaction energy components for the accuracy of MM-GBSA prediction

In the next step of our study, we investigated the role of LIE components in ML-based MM-GBSA predictions. Specifically, we aimed to assess whether predictive models remain capable of accurately estimating MM-GBSA binding free energies when electrostatic and van der Waals interaction energies (i.e., LIE terms) are excluded. For this purpose, all models were trained without LIE descriptors.

**Fig. 6:**
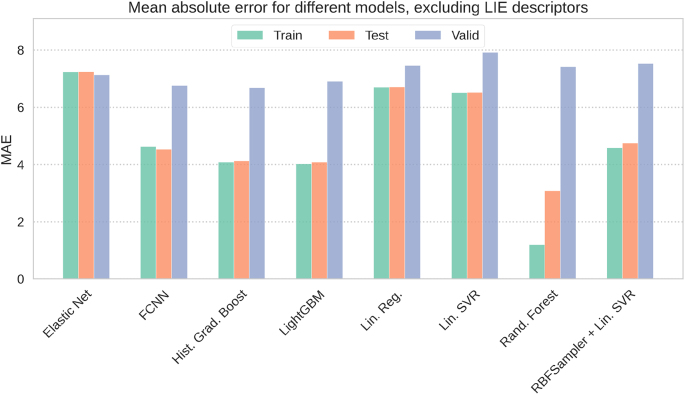
Comparison of the MAE metrics on train, test and validation sets for ML models tested in this study in the absence of LIE components. The values of MAE are given in units of kcal/mol.

Overall, the exclusion of LIE terms resulted in a noticeable decrease in predictive performance across all models, as reflected by higher MSE (Figure S12), higher MAE (Fig. 6) and lower $R^{2}$ values (Figure S13). The extent of this deterioration varied depending on the algorithm and was particularly pronounced for the validation set. The largest drops in accuracy were observed for LinearSVR and Linear Regression, consistent with the strong Pearson correlation coefficients of LIE terms with MM-GBSA energies. This highlights their key contribution to linear models. In contrast, more complex architectures such as FCNN, HistGradientBoost, and LightGBM were less affected, with FCNN achieving the highest validation performance even without LIE components ($R^{2}$ = 0.533), followed by HistGradientBoost ($R^{2}$ = 0.525) and LightGBM ($R^{2}$ = 0.491).

**Fig. 7:**
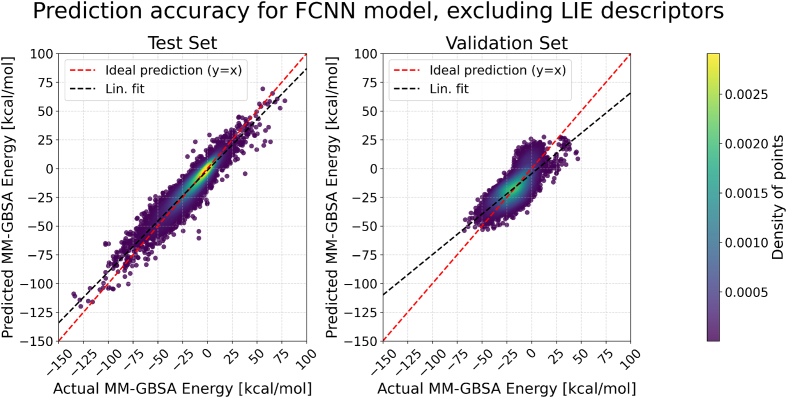
Comparison of actual and predicted MM-GBSA binding free energies for the FCNN model trained without LIE descriptors. Scatter plots show the prediction performance on the test set (left), and validation set (right). Color intensity represents the density of points, as estimated using 2D kernel density estimation (KDE). The red dashed line indicates the ideal prediction (y = x). (For interpretation of the references to colour in this figure legend, the reader is referred to the web version of this article.)

A detailed inspection of predicted versus actual MM-GBSA values revealed that FCNN (Fig. 7) produced predictions with a noticeably broader spread around the ideal y = x line, particularly underestimating affinities in the –25 to 25 kcal/mol range. Hist. Grad. Boost (Figure S14) exhibited an even wider dispersion of residuals, with systematic overestimation of weak binders—a trend also observed in LightGBM, though less pronounced (Figure S15). In Rand. Forest, weak binders were overestimated, while strong binders were underestimated (Figure S16). Linear Regression systematically overestimated all predictions (Figure S17) and LinearSVR displayed large deviations and overestimation of weak binders but offered slightly improved accuracy for the strongest binders (Figure S18). LinearSVR with RBFSampler again showed increased deviations and stronger overestimation of weak binders compared to the same model with LIE terms included (Figure S19). Finally, for ElasticNet, weak binders were systematically overestimated (Figure S20).

**Fig. 8:**
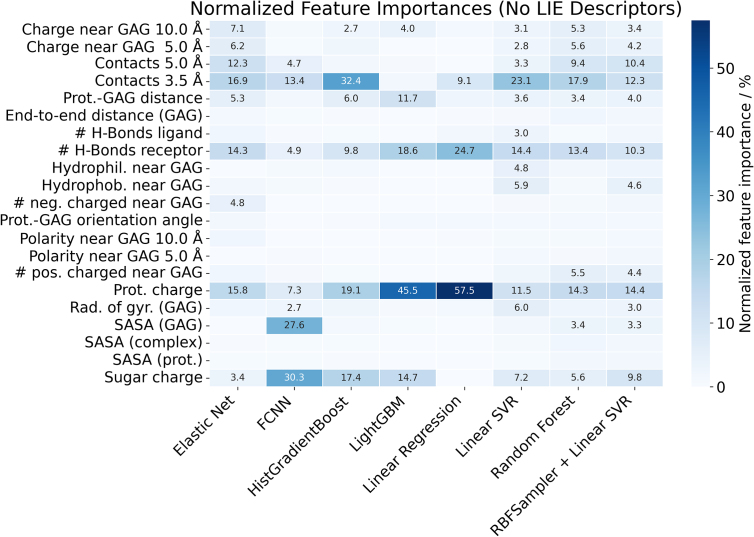
Normalized features importance for all ML models trained excluding LIE components.

When LIE components are removed, the distribution of feature importance changes substantially, and model-specific differences become more pronounced (Fig. 8). In Linear Regression, protein charge and the number of receptor-donor hydrogen bonds gain significance, consistent with their direct correlations with MM-GBSA. In ElasticNet, HistGradientBoost, LinearSVR, and Rand. Forest, short-range contacts become the dominant feature, followed by receptor-donor hydrogen bonds and protein charge. For the FCNN model, the two most influential descriptors shift to GAG charge and SASA, again emphasizing the role of electrostatics. In LinearSVR with RBFSampler, the absence of LIE highlights protein charge, short- and medium-range contacts, receptor-donor hydrogen bonds, and GAG charge, suggesting a more balanced reliance on both electrostatic and structural descriptors. LightGBM remains the most balanced model also in this setting, with the most relevant features including (pro)cathepsin–GAG distance, GAG charge, SASA, radius of gyration, and protein charge.

Taken together, these results demonstrate that LIE descriptors are critical for achieving high accuracy in ML-based MM-GBSA predictions. Their absence most severely impacts linear models, while more advanced non-linear architectures, particularly FCNN, retain a reasonable level of predictive power, highlighting their robustness in scenarios where LIE components are unavailable.

#### 3.3.4 Tuning neural network architecture and hyperparameters

In order to identify the most effective architecture of the FCNN, we performed a two-step hyperparameter optimization. In the first step, we systematically varied the number of hidden layers and the dropout rate. The results indicated that, in general, increasing the dropout rate led to a decline in predictive performance, particularly on the validation set (Table S1). The best performance was observed for the simplest architecture, with zero hidden layers and a dropout rate of 0.01, which showed the highest generalization capability to unseen data (Figure S21). Interestingly, for a dropout rate of 0.05, the negative effect of adding hidden layers on model performance was less pronounced, suggesting a partial regularizing effect at this dropout level. In contrast, models without any dropout exhibited a clear tendency to overfit the training data, especially as the network depth increased. These findings highlight that, in this particular case, simpler architectures with minimal regularization performed best, while deeper networks required stronger regularization to avoid overfitting.

In the next step, we evaluated FCNN models using a fixed dropout rate of 0.01 (selected based on the previous experiment), varying the number of hidden layers and testing different activation functions. The most accurate predictions were again obtained for the simplest model with zero hidden layers combined with the ReLU activation function (Figure S22). Comparable performance was also observed for models with zero hidden layers using ELU and LeakyReLU, indicating that these activation functions can perform similarly well in shallow network configurations (Table S2). In contrast, models using GELU and Sigmoid yielded slightly less accurate predictions, although still within a reasonable range. The Tanh activation function consistently led to the least accurate results across tested configurations. These results suggest that activation functions with linear or piecewise-linear characteristics, such as ReLU and LeakyReLU, may be better suited for MM-GBSA energy prediction than strongly non-linear alternatives. Consistent with the previous findings, we again observed that increasing the number of hidden layers led to reduced predictive accuracy, regardless of the activation function applied.

#### 3.3.5 Towards practical applications–single structure filtering, simulation-based ranking and minimum amount of data

**Fig. 9:**
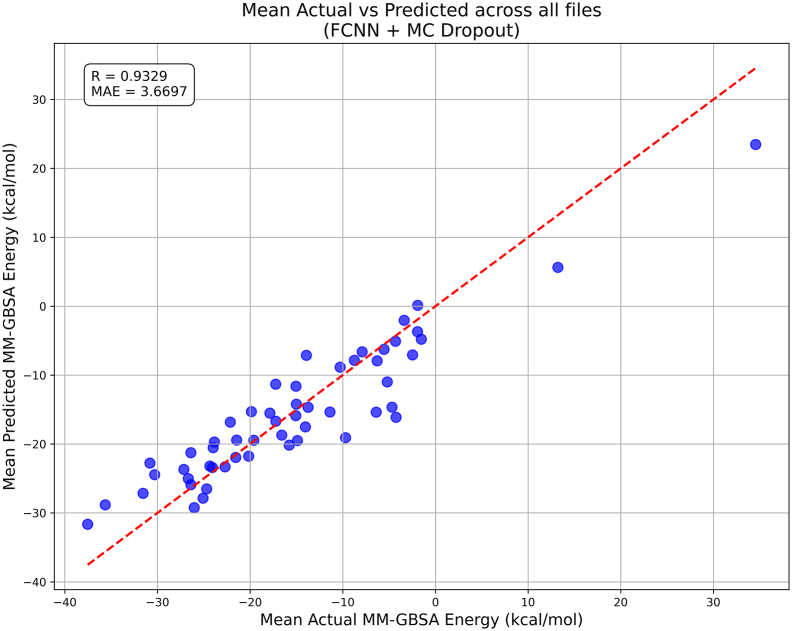
Comparison of actual and predicted mean MM-GBSA binding free energies for the FCNN model for cathepsin S-GAG MD simulations. The red dashed line indicates the ideal prediction (y = x). (For interpretation of the references to colour in this figure legend, the reader is referred to the web version of this article.)

Next, we evaluated the practical utility of the FCNN model. For this analysis, the validation dataset was split according to the MD simulations of cathepsin S–GAG complexes from which it originated. For each simulation, MM-GBSA energies were predicted using the most accurate FCNN model. The mean predicted energies were then compared with the mean calculated MM-GBSA energies. The correlation analysis revealed that the predicted averages closely reproduce the calculated values, with a Pearson correlation coefficient of 0.933 and a MAE of 3.670 kcal/mol (Fig. 9). These results indicate that the FCNN model can reliably predict mean MM-GBSA energies for entire trajectories, significantly reducing the computational cost of the analyses.

We further investigated whether the FCNN model can provide meaningful predictions using only the first frame from each simulation. Predicted MM-GBSA energies for the first frame were compared against the simulation-averaged calculated energies. This analysis showed moderate correlation (R = 0.644, MAE = 8.584 kcal/mol), indicating that initial structures alone are less accurate for precise ranking of the complexes (Figure S23). Nevertheless, these results suggest that the model can be used as an initial filter to identify potentially weak binders, allowing subsequent MD simulations to focus on the most promising candidates. Overall, these findings demonstrate that the FCNN model is not only effective in reproducing average MM-GBSA values but also valuable for the preliminary classification of strong and weak binders.

We also investigated the effect of reducing the amount of training data on the prediction accuracy of the trained FCNN model. To this end, we systematically skipped data points in our training data set and trained FCNN models from scratch with decreasing number of data points. The results of all metrics ($R^{2}$, MAE, and MSE) in the different data sets (training, test and validation) with decreasing amount of training data are shown in Figures S24, S25 and S26. We found that the amount of data may be reduced to as little as 2 % of the total amount of collected data, without substantial decrease in the prediction accuracy. Even 1 % of the data–which corresponds to ca. 8400 data points–still yields tolerable accuracy, i.e., $R^{2}=0.602$ for the validation set. From this investigation, we conclude that at least multiple thousand data points are necessary to provide decent accuracy for a prediction task of the complexity. In this context, it needs to be remarked that MD simulation data is generally time correlated. Therefore, by systematically skipping data points, to a certain extent (defined by the decorrelation time in the simulation), redundant data points are skipped. Whether the here-obtained number of necessary data points is transferable to other prediction tasks, is not straightforward to assess. With respect to this question, the following aspects need to be taken into account: diversity of the data set to be evaluated (which includes the noise level in the data), and also the complexity of the input data structure. Furthermore, the dependency of the accuracy on the amount of training data also varies for different machine learning models. For example deep neural networks are known to be particularly data-hungry [66].

Lastly, we investigated the efficiency of MM-GBSA predictions using the FCNN model by comparing the time required to obtain descriptors and predict MM-GBSA energies with the time needed to calculate the same energies using the standard approach. The results indicate that, in general, MM-GBSA predictions are approximately five times faster than direct calculations (Figure S27). The distributions of frames versus analysis time show that the average frames-per-second (fps) ratio for the prediction approach is ∼0.74, whereas for the standard calculations it is ∼0.15. This speedup becomes more pronounced with increasing size of the dataset, highlighting the computational advantage of ML-based approaches for high-throughput analyses. All computations were performed locally on the same workstation under equivalent conditions, and the MM-GBSA protocol was not parallelised. These findings highlight that MM-GBSA predictions can serve as not only a reliable but also an efficient alternative to conventional calculations.

## 4 Conclusion

Given the scarcity of experimental and structural data on (pro)cathepsin–GAG complexes, the development of novel *in silico* strategies provides a promising avenue for exploring these systems at the atomic level. Because experimental methods are costly and complex, ML-based approaches may offer valuable insights that would otherwise be difficult to obtain. In this study, we trained eight ML models to predict MM-GBSA binding free energies and demonstrated their potential as efficient tools for comparing the stability of (pro)cathepsin–GAG complexes.

The analysis of correlations between structural descriptors and MM-GBSA binding free energies revealed that the most influential features are electrostatic in nature. These include the electrostatic component of LIE, the electrostatic potential on the protein surface, and the number of positively charged residues in the (pro)cathepsin. This further emphasizes the electrostatic character of protein–GAG interactions. Features associated with polarity, such as the number of hydrophilic and hydrophobic residues near the GAG molecule and the polarity of the (pro)cathepsin surface within 5 Å of the GAG, were identified as the second most important group of descriptors. In contrast, purely structural or geometrical features (e.g., the radius of gyration, end-to-end distance of the GAG, or SASA values for the protein, GAG, or complex) exhibited the weakest correlations with binding free energies. These findings not only highlight the electrostatically driven nature of protein–GAG binding but also provide a valuable framework for rational feature selection in future ML-based approaches to glycan–protein interactions.

Among the tested models, the best performance was achieved by the FCNN, which delivered the highest accuracy on the validation set. Comparable predictions were obtained with gradient-boosting models, although these showed slight overfitting to the training data. The issue was even more pronounced in the Rand. Forest model. Classical regression models such as Linear Regression and LinearSVR ranked behind the boosting models but achieved comparable levels of accuracy. However, they struggled to correctly identify the most and least stable complexes. This behaviour can be attributed to the intrinsic nature of the features used. In both very stable and very unstable complexes the differences encoded in the descriptors can be subtle or even saturate, especially for short-range contacts, electrostatic contributions, and cooperative hydrogen-bond networks. As a result, the models may find it difficult to learn sufficiently distinct patterns for the extremes, leading to overestimation of strong binders and underestimation of weak ones. Interestingly, the addition of RBFSampler to LinearSVR decreased its accuracy. ElasticNet performed the worst among all tested models. While the FCNN is recommended as the primary model due to its overall highest accuracy, Linear Regression remains a useful alternative if prioritizing model simplicity and interpretability over maximal predictive performance.

Hyperparameter optimization of the FCNN indicated that models with a small dropout rate (0.01) and minimal architectural complexity (i.e., no hidden layers) yielded the most accurate predictions. Further testing of activation functions revealed that ReLU was the most effective for (pro)cathepsin–GAG complexes, although ELU and LeakyReLU provided comparable results.

In particular, we investigated the importance of LIE components for the prediction accuracy of MM-GBSA results. We found that classical regression models, such as Linear Regression and LinearSVR, were most affected by the absence of these energetic descriptors, resulting in a substantial loss of accuracy. Advanced models based on decision trees and neural networks also showed performance drops, although to a lesser extent. This is likely because Linear Regression and LinearSVR rely heavily on the electrostatic LIE component, which is highly correlated with MM-GBSA energies. In its absence, they attempted to compensate using descriptors related to protein charge, the number of receptor–donor hydrogen bonds, and the number of short-range contacts. More advanced models, such as the FCNN, were better able to compensate for the absence of electrostatic LIE components using alternative electrostatics-related descriptors, such as GAG charge and SASA.

Another goal of this study was to estimate the minimal dataset size required to train an FCNN capable of providing accurate predictions. The results showed that an accurate FCNN model can be trained with as little as 2 % of the initial dataset which in this study corresponds to approximately 17,000 data points. Further reductions, however, led to dramatic decrease in prediction accuracy on the validation set. This observation suggests that, for predicting complex properties such as binding affinity, a dataset comprising at least several thousand data points may be necessary to achieve reliable predictions. Generally, our results indicate good predictions for the validation set. Nevertheless, the transferability of the model may be further improved, if even more diverse datasets–in terms of (pro)cathepsin-GAG combinations–were available.

Finally, we highlighted the practical utility of the trained FCNN model. The results indicate that mean predicted MM-GBSA energies correlate strongly with their mean calculated counterparts, suggesting that this ML approach can serve as a computationally efficient alternative to standard MM-GBSA calculations in MD trajectory analyses. Moreover, a significant, yet moderate, correlation was observed between the predicted MM-GBSA energies for the first frames of each trajectory and the mean calculated MM-GBSA energies across the trajectories. While predictions based solely on the first frames may not suffice to accurately rank all complexes, they may still provide a useful tool for distinguishing strong from weak binders. This in turn could help prioritize MD simulations for selected complexes, thereby saving computational resources.

Overall, our results demonstrate that machine learning approaches can be effectively applied to study the energetics of (pro)cathepsin–GAG complexes. Nevertheless, there remains considerable potential for further improvement: The inclusion of novel GAG-specific descriptors may enhance prediction accuracy. Besides that, since the current model is trained on a limited set of (pro)cathepsin–GAG complexes, extending the dataset to include a wider variety of protein–GAG systems would likely enhance its ability to generalize to other protein classes. Ultimately, this study provides a promising proof of concept for applying ML-based models to GAG-containing systems. This is particularly impactful, since protein–GAG binding is inherently complex and challenging to characterize with conventional computational methods.

## CRediT authorship contribution statement

**Krzysztof K. Bojarski:** Writing – review & editing, Writing – original draft, Visualization, Validation, Methodology, Investigation, Funding acquisition, Formal analysis, Data curation, Conceptualization. **Patrick K. Quoika:** Writing – review & editing, Writing – original draft, Validation, Methodology, Investigation, Formal analysis. **Martin Zacharias:** Writing – review & editing, Writing – original draft, Supervision, Formal analysis.

## Declaration of competing interest

The authors declare that they have no known competing financial interests or personal relationships that could have appeared to influence the work reported in this paper.

## Data Availability

The data supporting this study are openly available in the Zenodo repository (https://doi.org/10.5281/zenodo.16980049). The repository contains scripts for calculating structural descriptors of (pro)cathepsin–GAG complexes, as well as scripts for FCNN model training and prediction. In addition, it provides the calculated structural and energetic descriptors for the training, testing, and validation sets used in this work.
