# Supplementary material for: Machine learning models for prediction of (Pro)cathepsin–glycosaminoglycan binding free energies based on molecular structure
Source: Comput Struct Biotechnol J. 2025 Dec 8;31:61–73. doi: 10.1016/j.csbj.2025.11.059 (PMC12771359; doi:10.1016/j.csbj.2025.11.059)
Supplement: Multimedia Component 1 [file mmc1.pdf]

Machine Learning Models for Prediction of (Pro)cathepsin–Glycosaminoglycan Binding Free Energies Based on Molecular Structure

Krzysztof K. Bojarski*[1,2], Patrick K. Quoika[2], and Martin Zacharias[2]

[1]Department of Physical Chemistry, Gdansk University of Technology, Narutowicza 11/12, Gdansk, Poland
[2]Center for Functional Protein Assemblies, Technical University of Munich, Ernst-Otto-Fischer-Straße 8, Garching, Germany

Supplementary Information

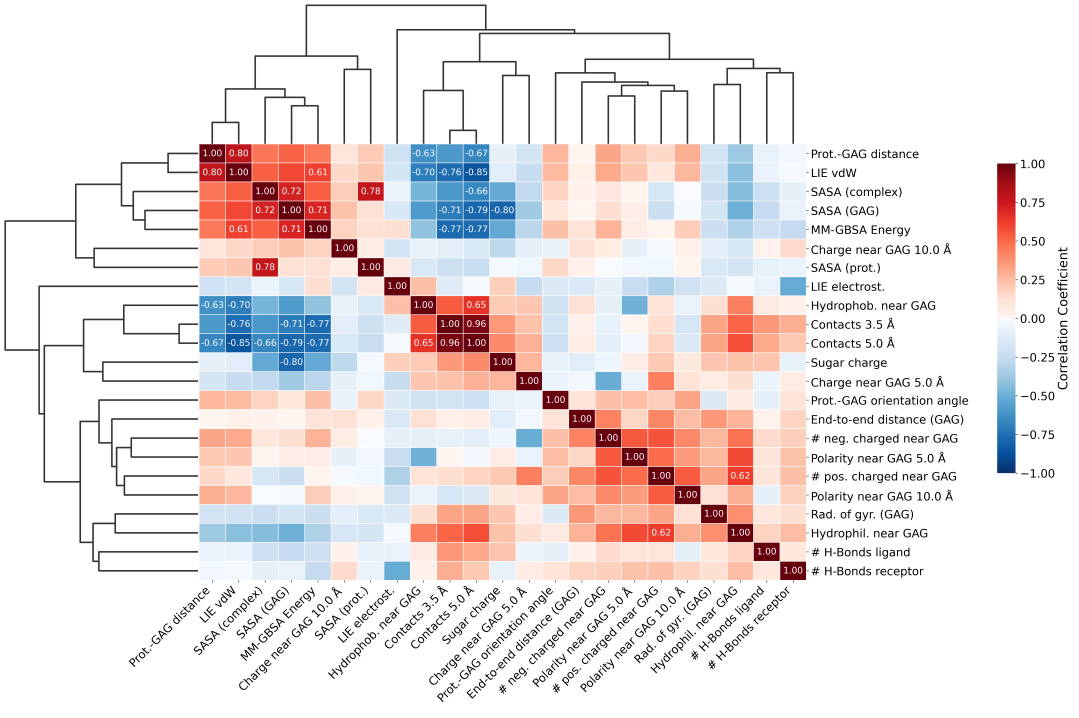

Figure S1: Correlation matrix of features within the validation set describing (pro)cathepsin–GAG interactions, with hierarchical clustering of features.

*Corresponding author: krzysztof.bojarski@pg.edu.pl

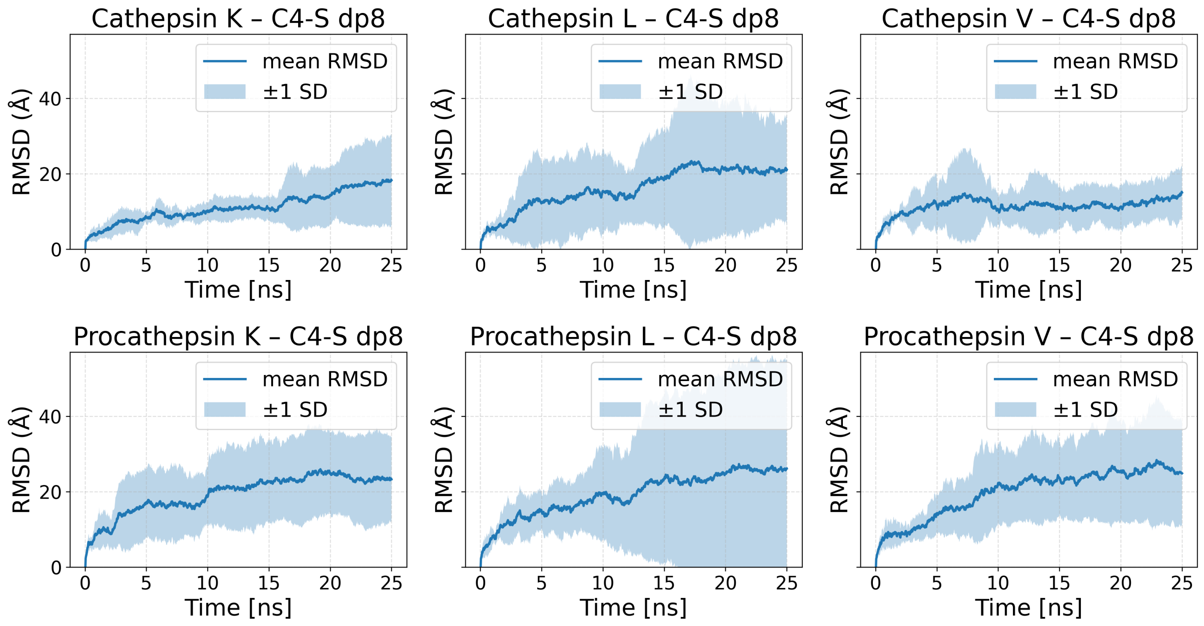

Figure S2: Averaged RMSD profiles with standard deviations for C4-S (dp8) with (pro)cathepsins K, L and V over six MD simulations starting from different binding poses

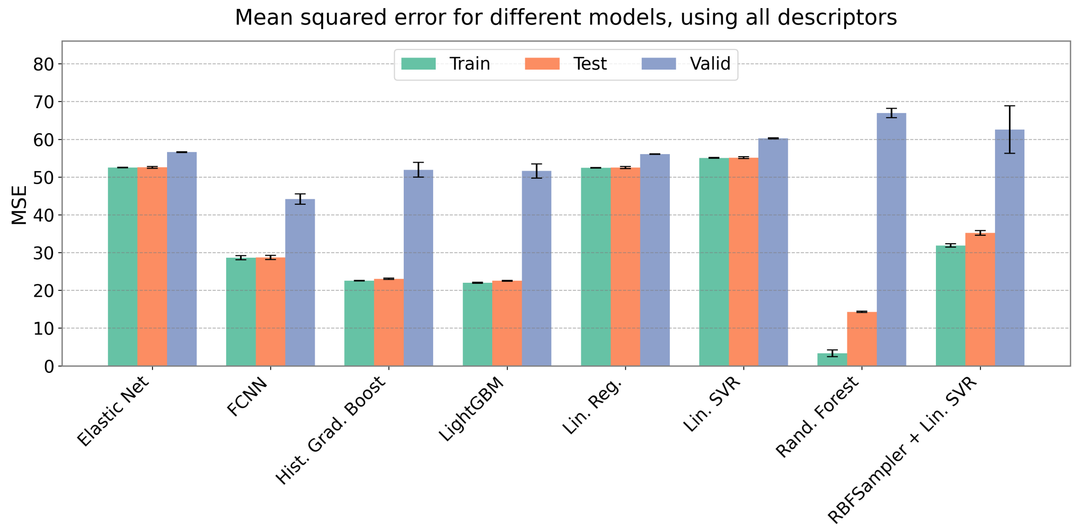

Figure S3: Comparison of the MSE metrics on Train, Test and Validation sets for ML models tested in this study.

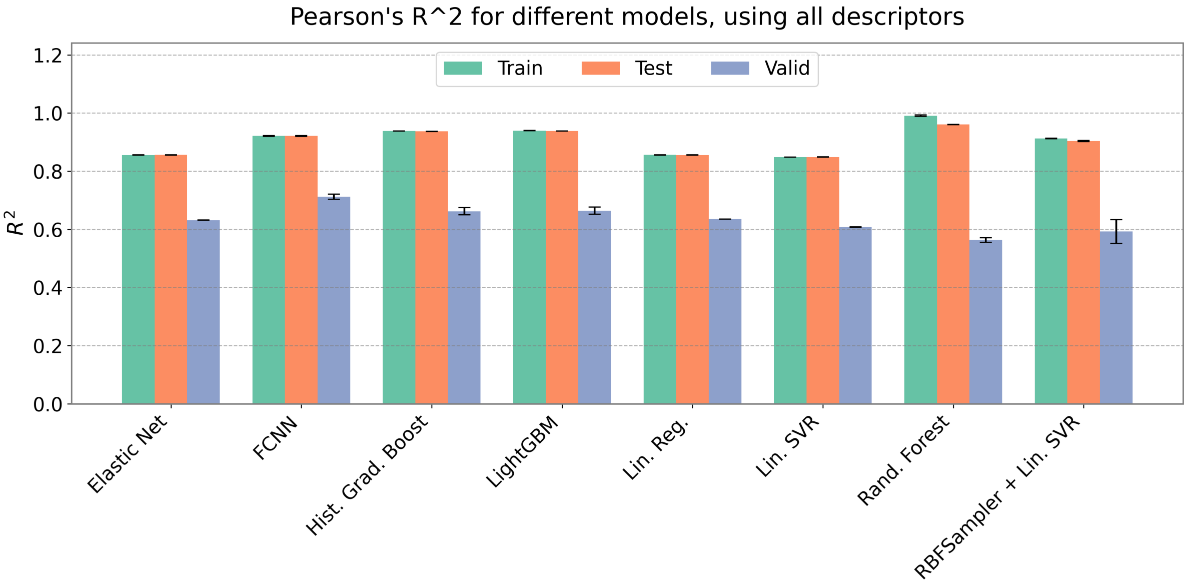

Figure S4: Comparison of the R^2 metrics on Train, Test and Validation sets for ML models tested in this study.

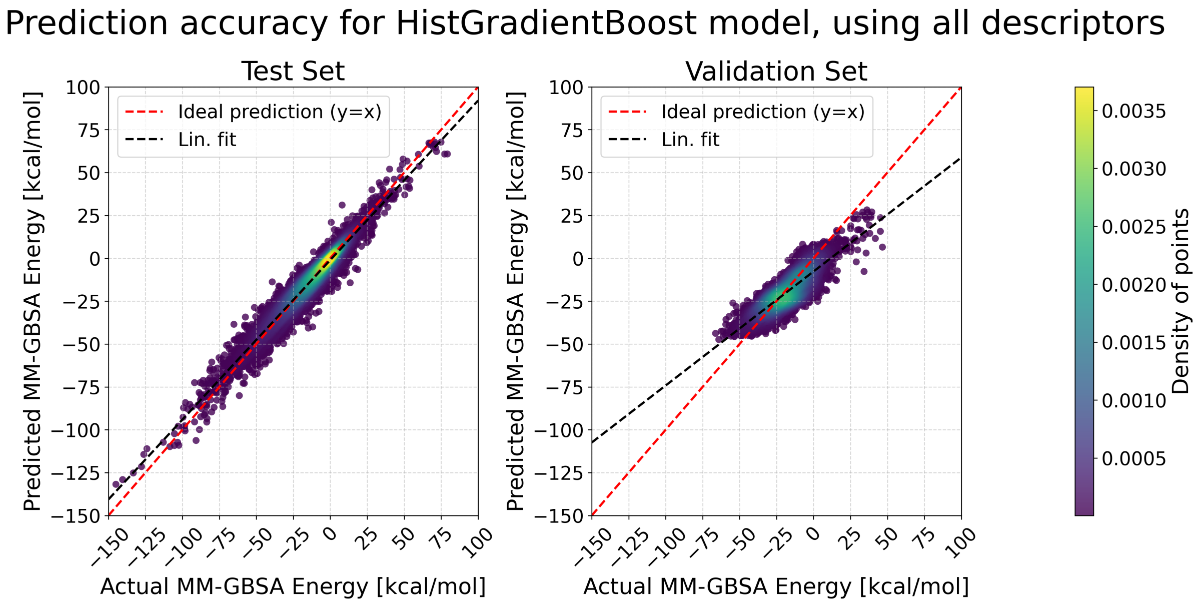

Figure S5: Comparison of actual and predicted MM-GBSA binding free energies for the HistGradientBoost model. Scatter plots show the prediction performance on the test set (left), and validation set (right). Color intensity represents the density of points, as estimated using 2D Kernel Density Estimation (KDE). The red dashed line indicates the ideal prediction (y = x).

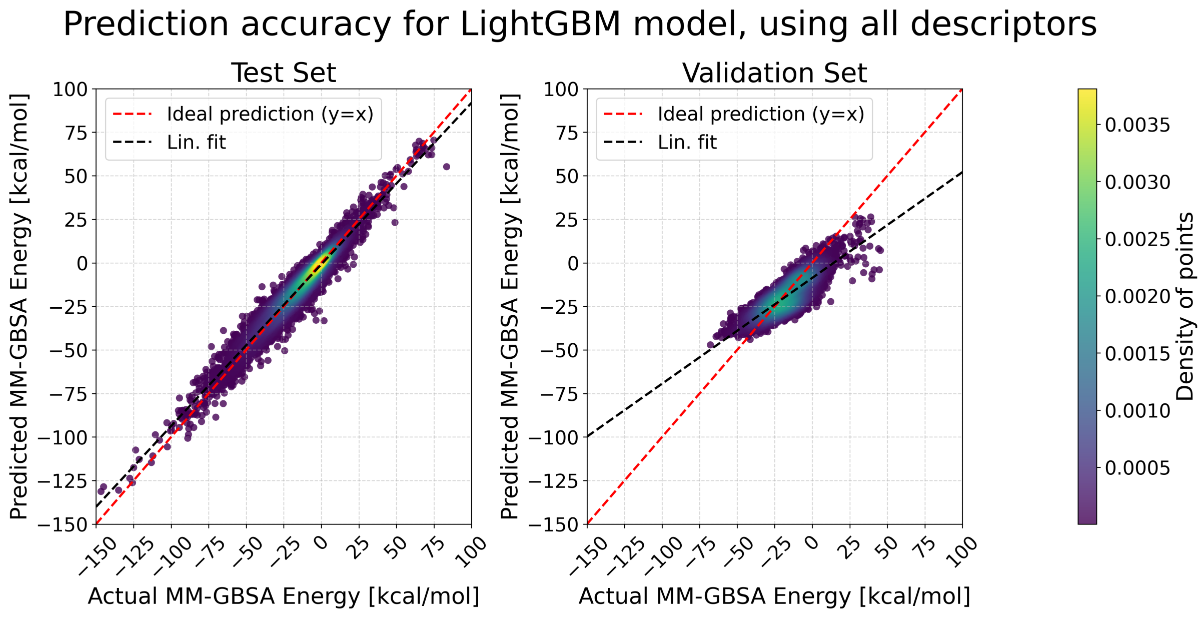

Figure S6: Comparison of actual and predicted MM-GBSA binding free energies for the LightGBM model. Scatter plots show the prediction performance on the test set (left), and validation set (right). Color intensity represents the density of points, as estimated using 2D Kernel Density Estimation (KDE). The red dashed line indicates the ideal prediction (y = x).

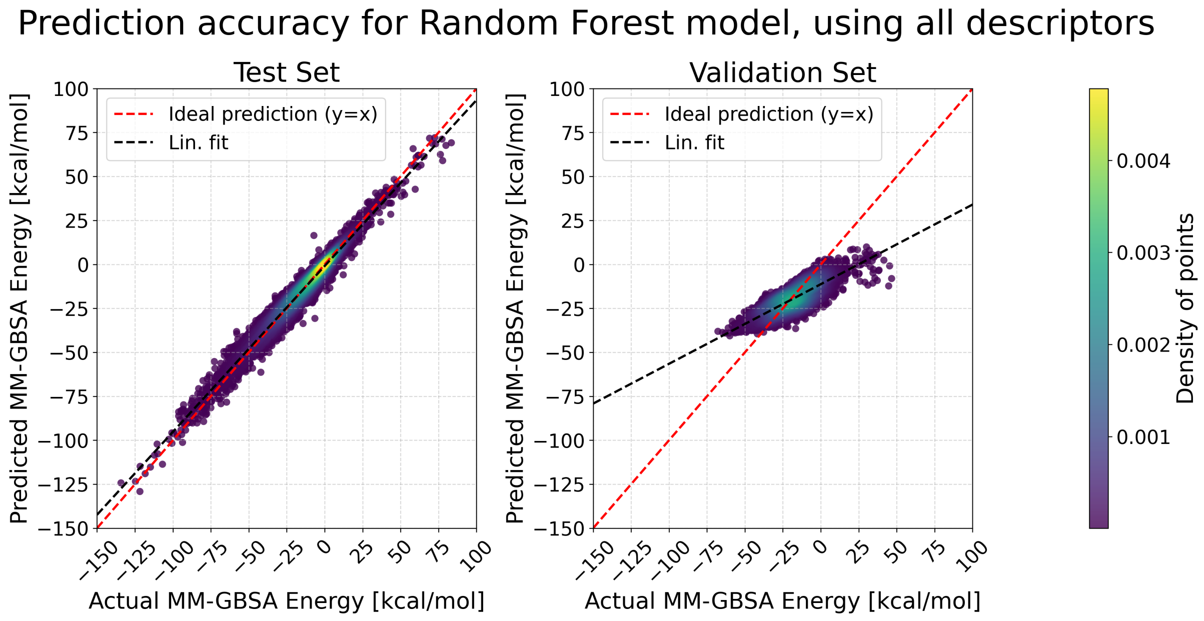

Figure S7: Comparison of actual and predicted MM-GBSA binding free energies for the Random Forest model. Scatter plots show the prediction performance on the test set (left), and validation set (right). Color intensity represents the density of points, as estimated using 2D Kernel Density Estimation (KDE). The red dashed line indicates the ideal prediction (y = x).

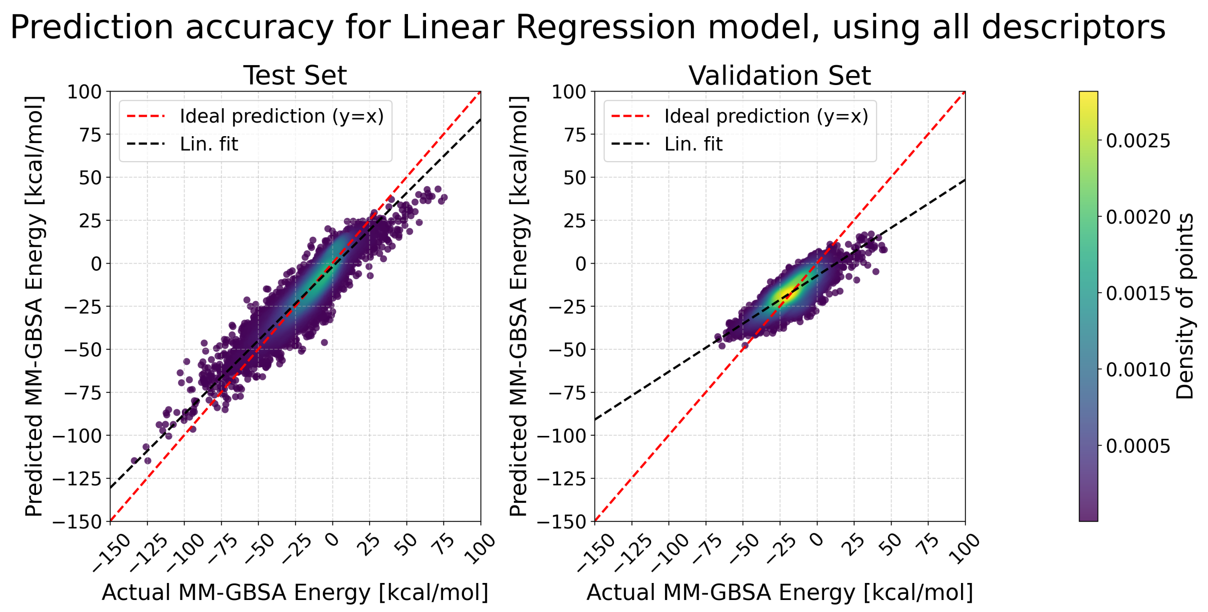

Figure S8: Comparison of actual and predicted MM-GBSA binding free energies for the Linear Regression model. Scatter plots show the prediction performance on the test set (left), and validation set (right). Color intensity represents the density of points, as estimated using 2D Kernel Density Estimation (KDE). The red dashed line indicates the ideal prediction (y = x).

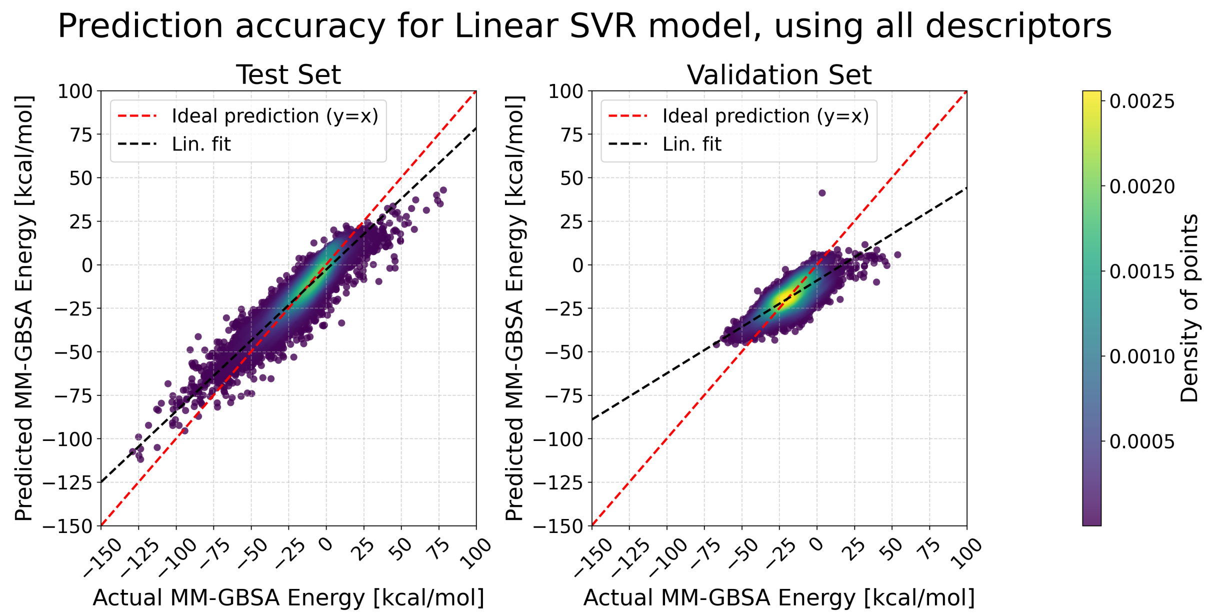

Figure S9: Comparison of actual and predicted MM-GBSA binding free energies for the LinearSVR model. Scatter plots show the prediction performance on the test set (left), and validation set (right). Color intensity represents the density of points, as estimated using 2D Kernel Density Estimation (KDE). The red dashed line indicates the ideal prediction (y = x).

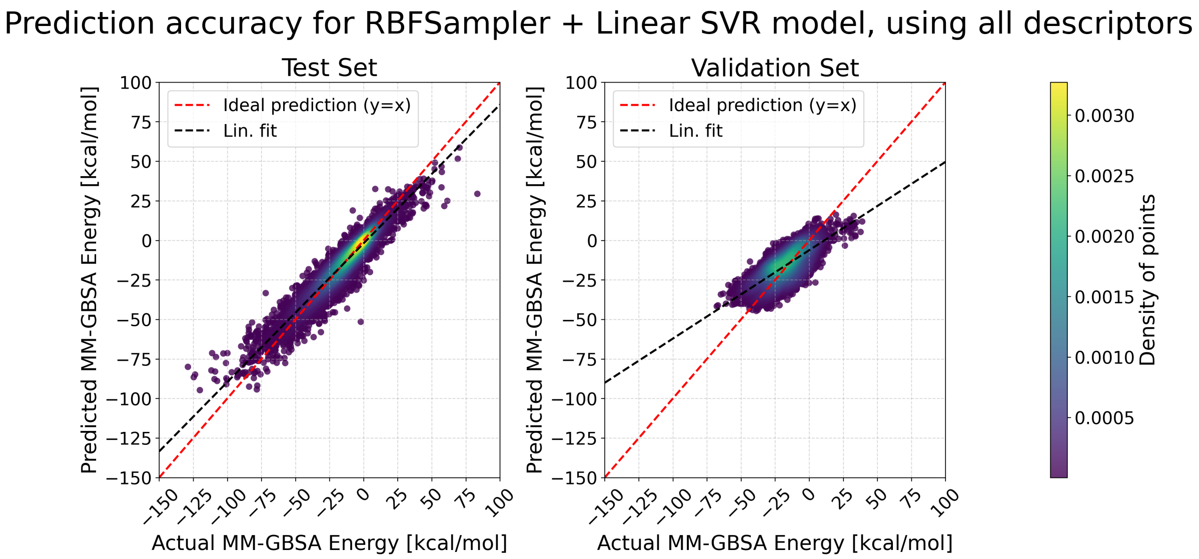

Figure S10: Comparison of actual and predicted MM-GBSA binding free energies for the LinearSVR model with RBFSampler. Scatter plots show the prediction performance on the test set (left), and validation set (right). Color intensity represents the density of points, as estimated using 2D Kernel Density Estimation (KDE). The red dashed line indicates the ideal prediction (y = x).

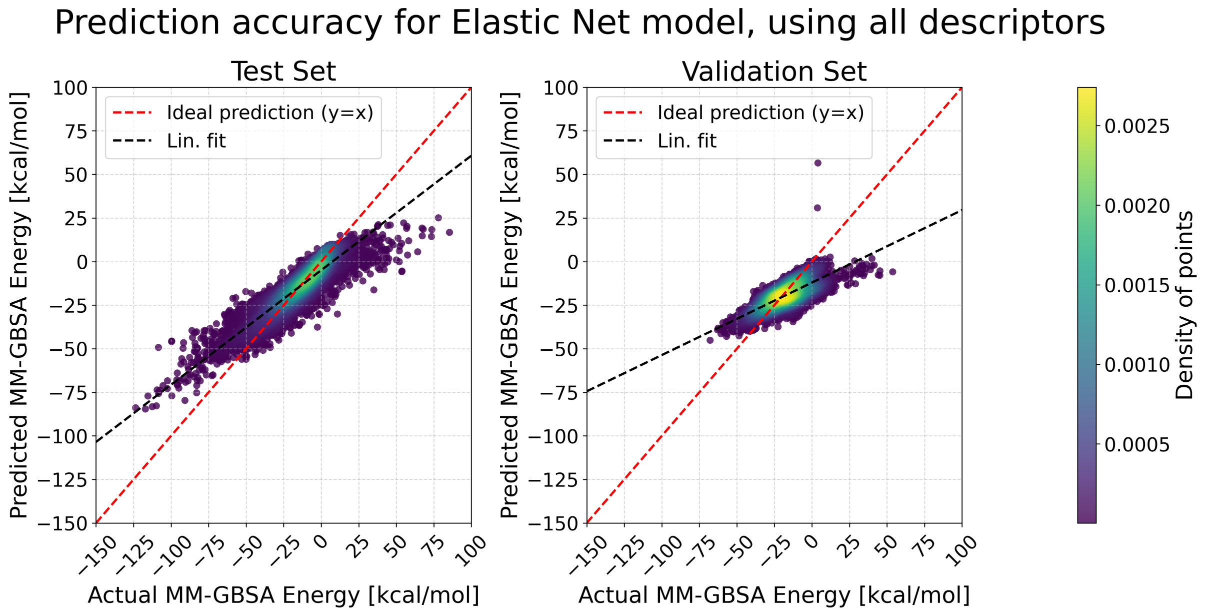

Figure S11: Comparison of actual and predicted MM-GBSA binding free energies for the ElasticNet model. Scatter plots show the prediction performance on the test set (left), and validation set (right). Color intensity represents the density of points, as estimated using 2D Kernel Density Estimation (KDE). The red dashed line indicates the ideal prediction (y = x).

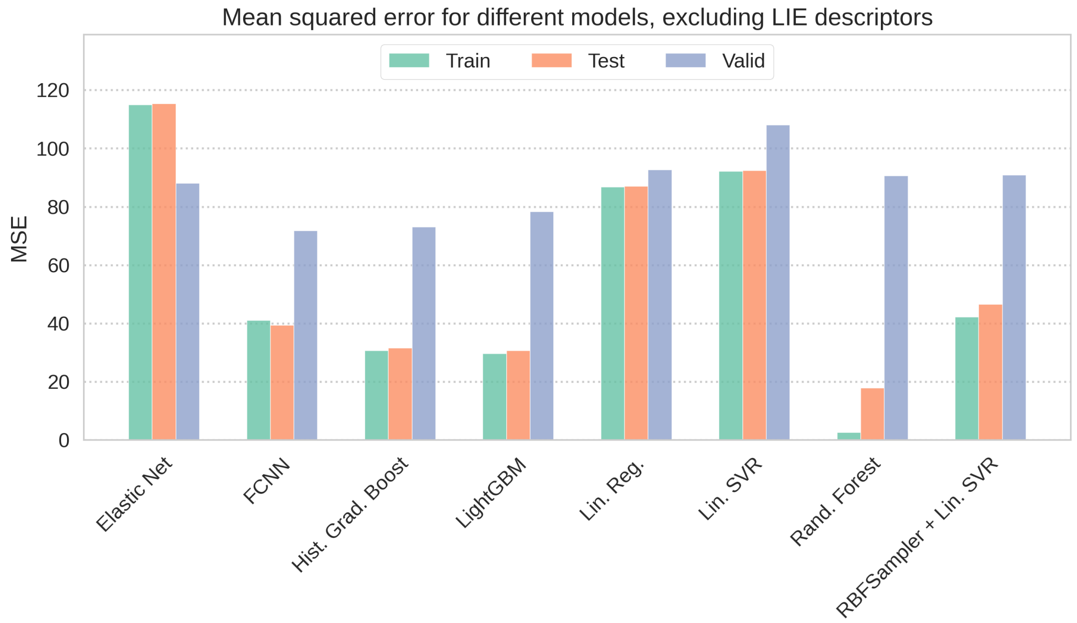

Figure S12: Comparison of the MSE metrics on Train, Test and Validation sets for ML models tested in this study in the absence of LIE components.

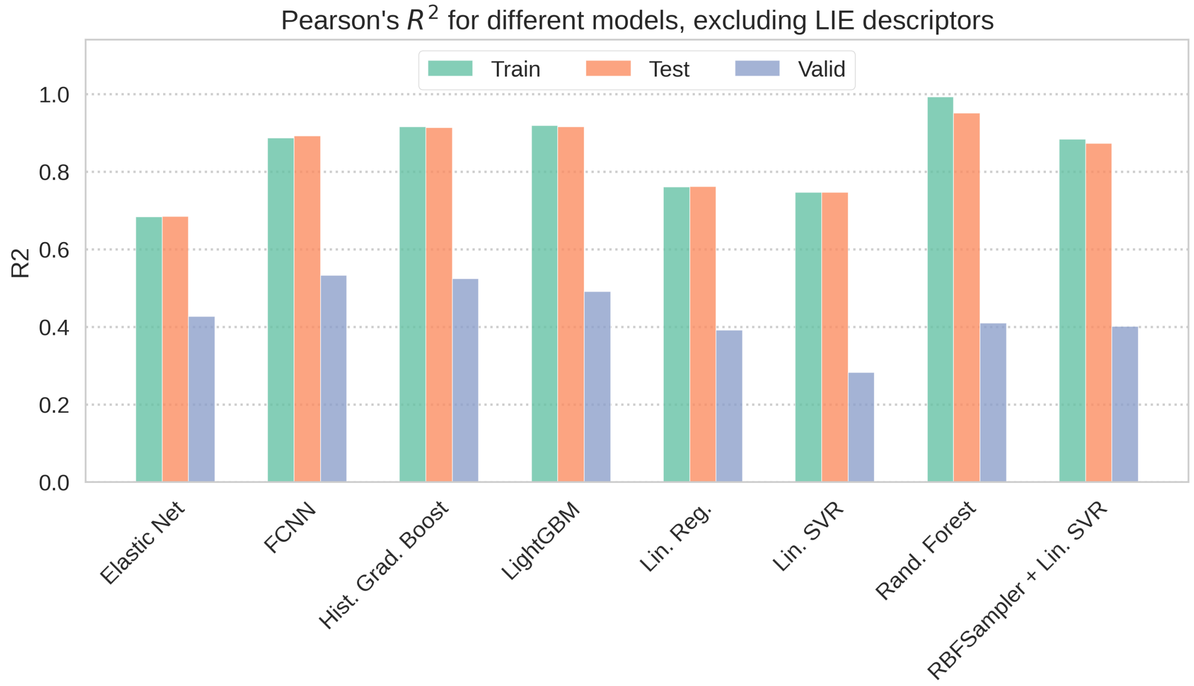

Figure S13: Comparison of the R^2 metrics on Train, Test and Validation sets for ML models tested in this study in the absence of LIE components.

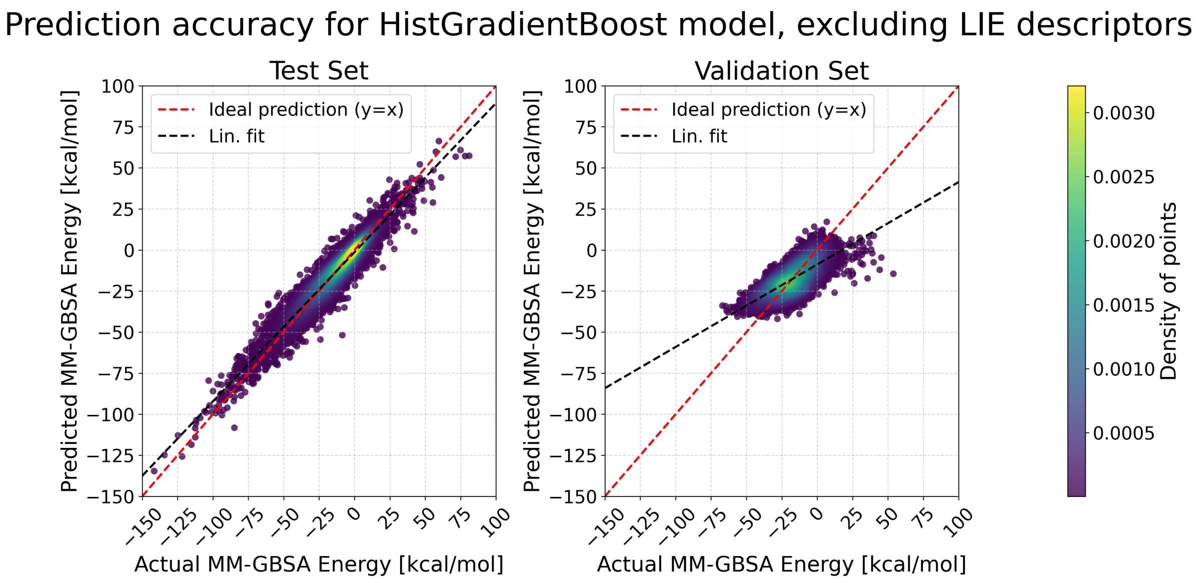

Figure S14: Comparison of actual and predicted MM-GBSA binding free energies for the HistGradientBoost model trained without LIE descriptors. Scatter plots show the prediction performance on the test set (left), and validation set (right). Color intensity represents the density of points, as estimated using 2D Kernel Density Estimation (KDE). The red dashed line indicates the ideal prediction (y = x).

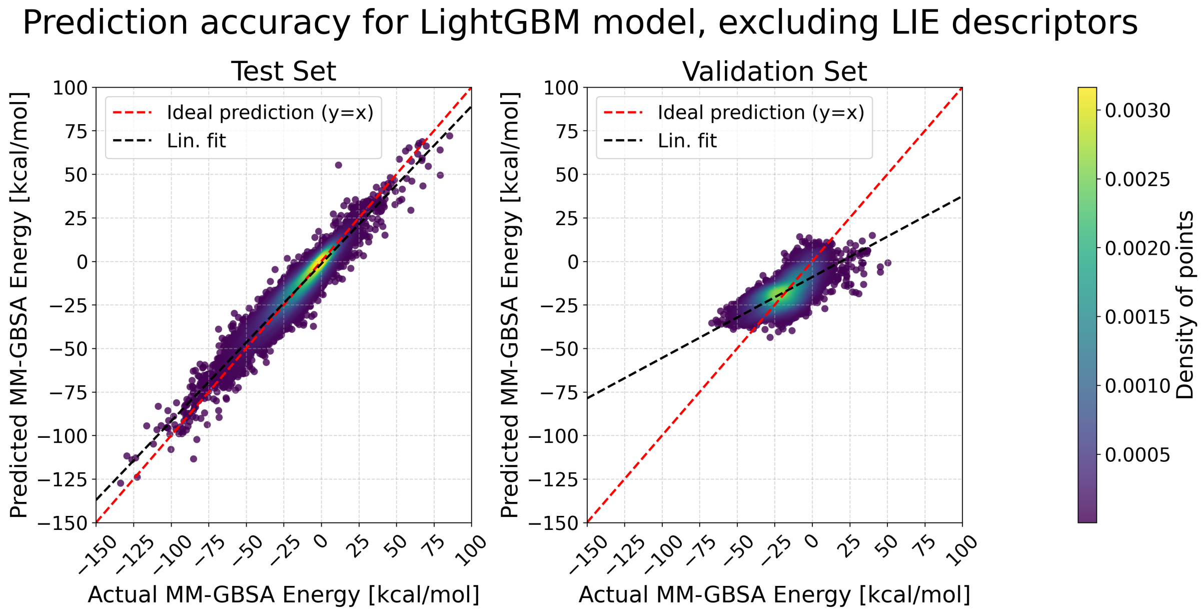

Figure S15: Comparison of actual and predicted MM-GBSA binding free energies for the LightGBM model trained without LIE descriptors. Scatter plots show the prediction performance on the test set (left), and validation set (right). Color intensity represents the density of points, as estimated using 2D Kernel Density Estimation (KDE). The red dashed line indicates the ideal prediction (y = x).

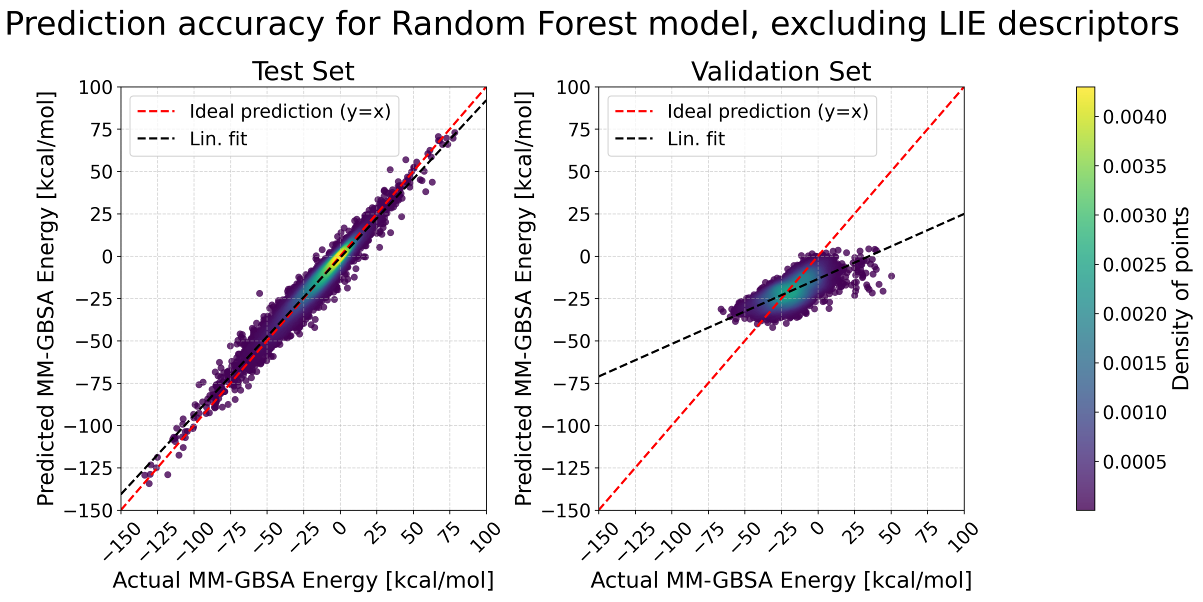

Figure S16: Comparison of actual and predicted MM-GBSA binding free energies for the Random Forest model trained without LIE descriptors. Scatter plots show the prediction performance on the test set (left), and validation set (right). Color intensity represents the density of points, as estimated using 2D Kernel Density Estimation (KDE). The red dashed line indicates the ideal prediction (y = x).

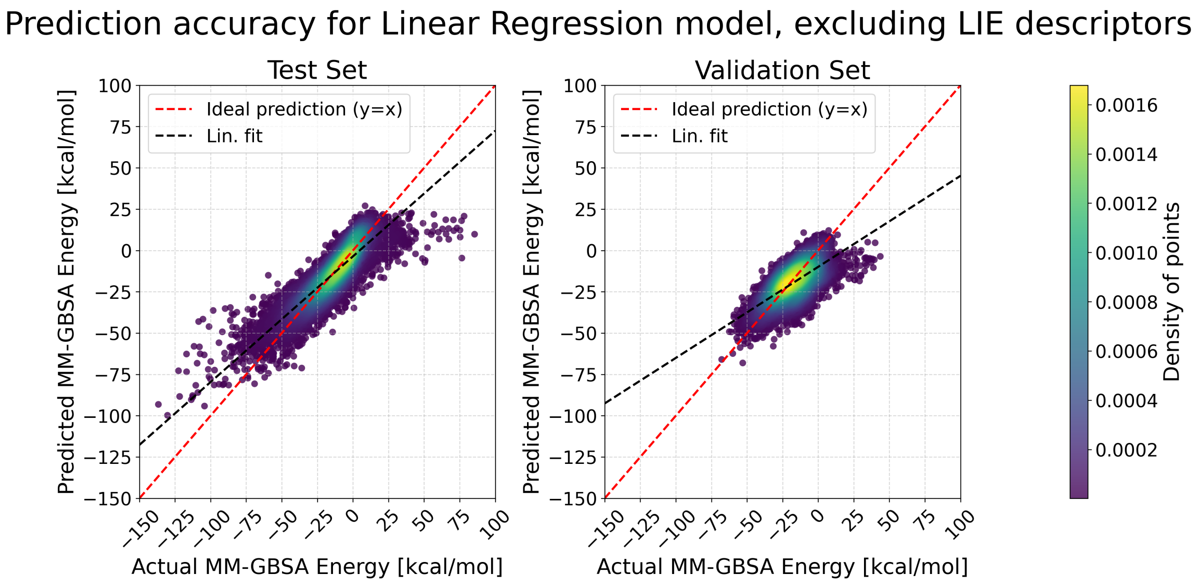

Figure S17: Comparison of actual and predicted MM-GBSA binding free energies for the Linear Regression model trained without LIE descriptors. Scatter plots show the prediction performance on the test set (left), and validation set (right). Color intensity represents the density of points, as estimated using 2D Kernel Density Estimation (KDE). The red dashed line indicates the ideal prediction (y = x).

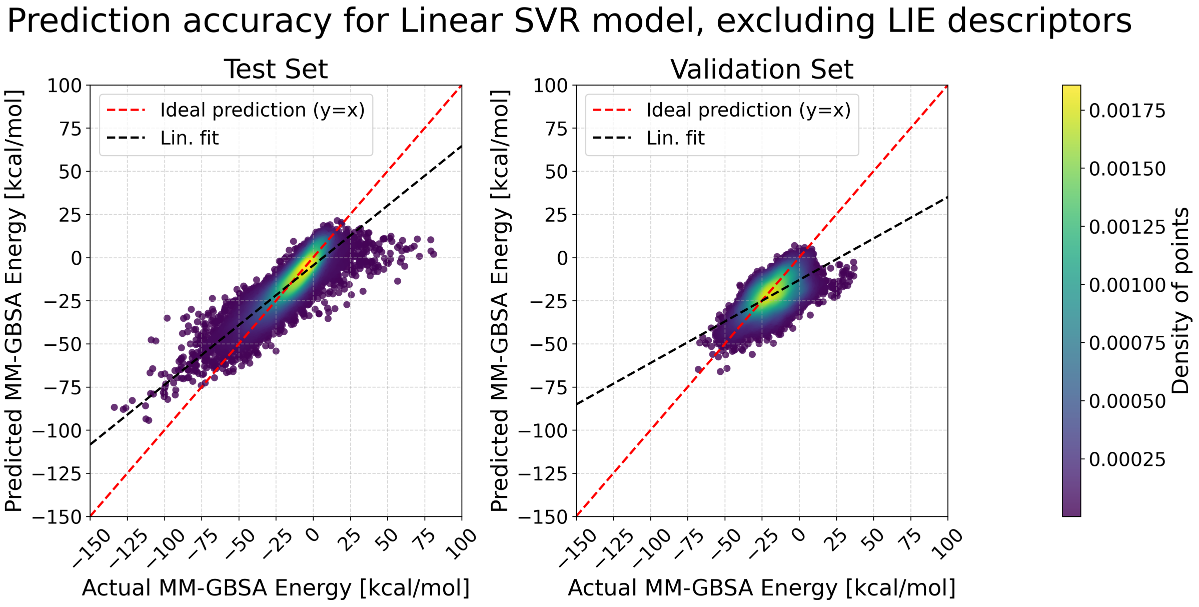

Figure S18: Comparison of actual and predicted MM-GBSA binding free energies for the LinearSVR model trained without LIE descriptors. Scatter plots show the prediction performance on the test set (left), and validation set (right). Color intensity represents the density of points, as estimated using 2D Kernel Density Estimation (KDE). The red dashed line indicates the ideal prediction (y = x).

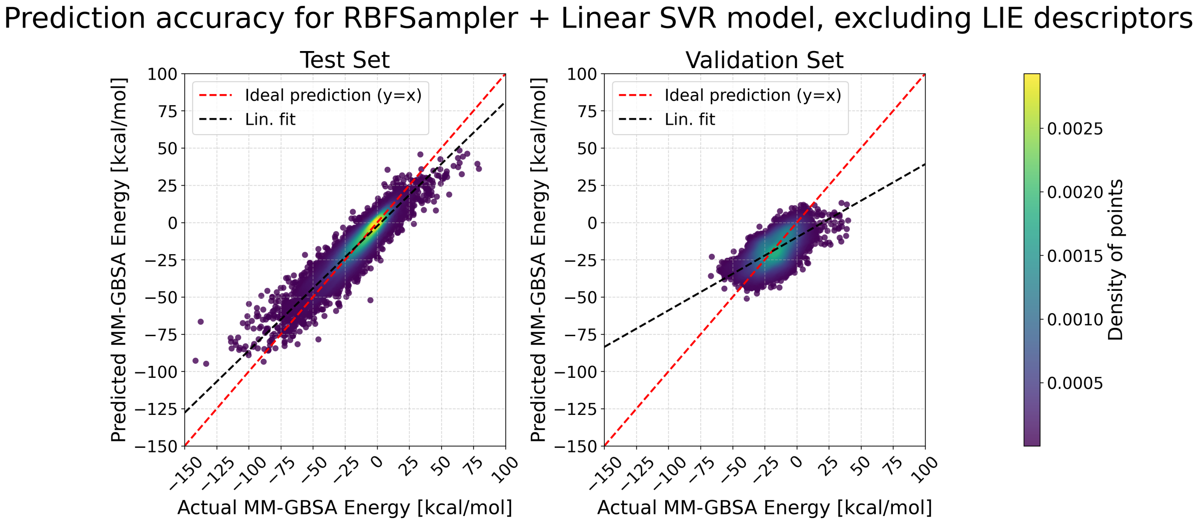

Figure S19: Comparison of actual and predicted MM-GBSA binding free energies for the LinearSVR model trained without LIE descriptors and with RBFSampler. Scatter plots show the prediction performance on the test set (left), and validation set (right). Color intensity represents the density of points, as estimated using 2D Kernel Density Estimation (KDE). The red dashed line indicates the ideal prediction (y = x).

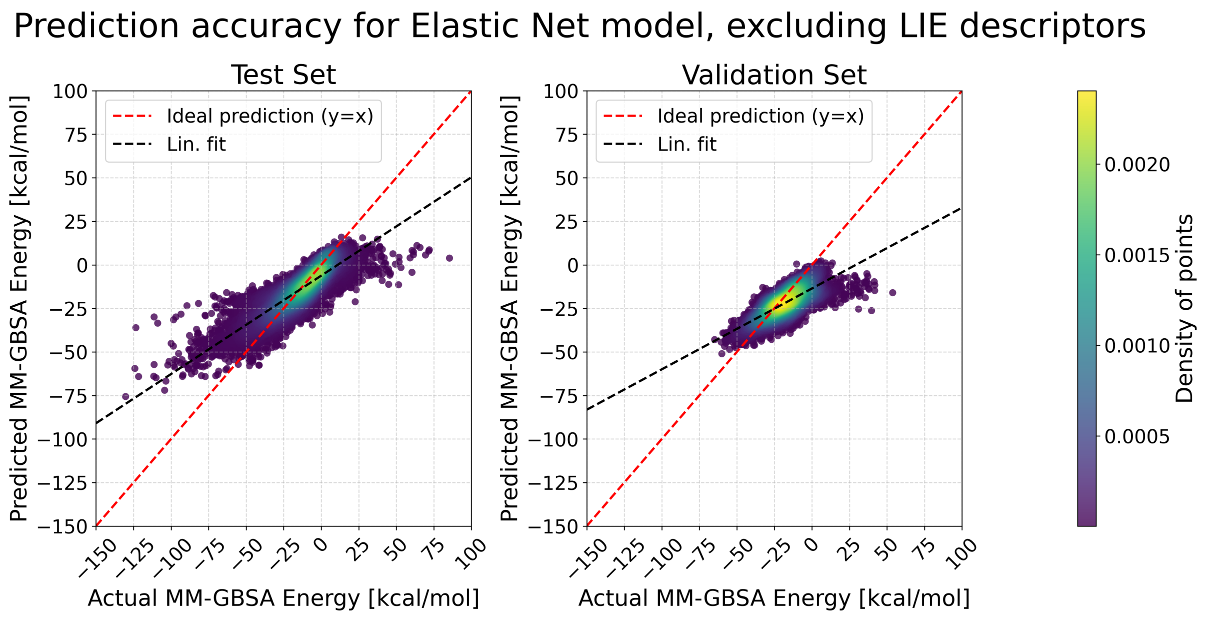

Figure S20: Comparison of actual and predicted MM-GBSA binding free energies for the ElasticNet model trained without LIE descriptors. Scatter plots show the prediction performance on the test set (left), and validation set (right). Color intensity represents the density of points, as estimated using 2D Kernel Density Estimation (KDE). The red dashed line indicates the ideal prediction (y = x).

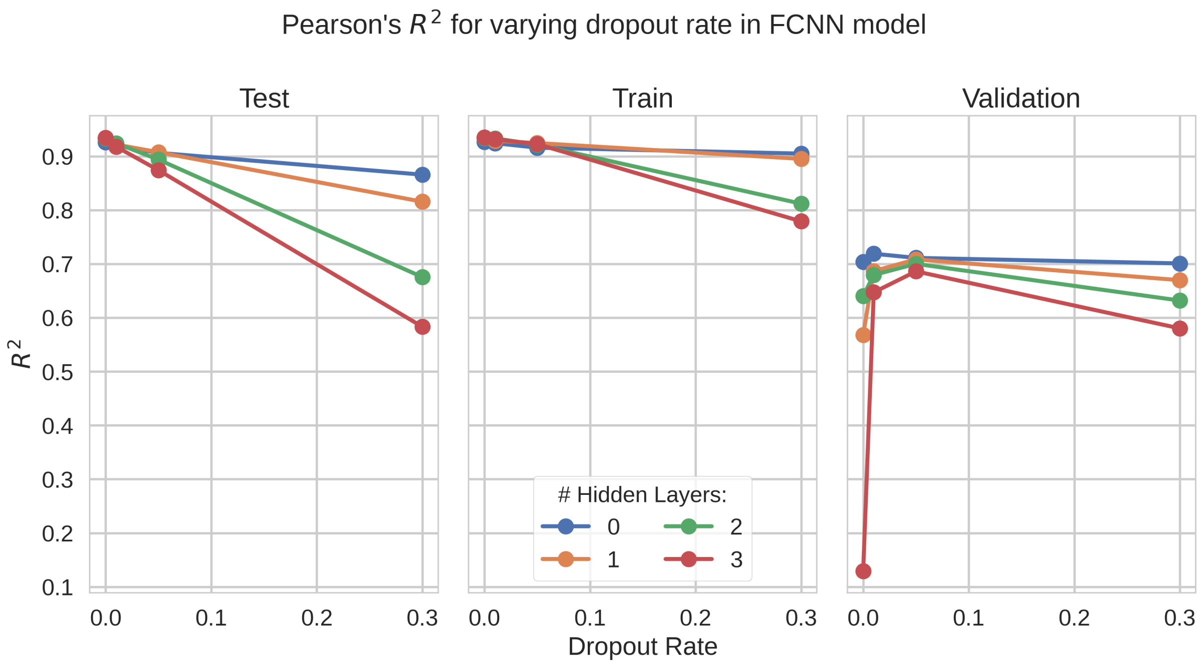

Figure S21: Comparison of the R^2 metrics on Train, Test and Validation sets for FCNN models with different dropout rates and different number of hidden layers.

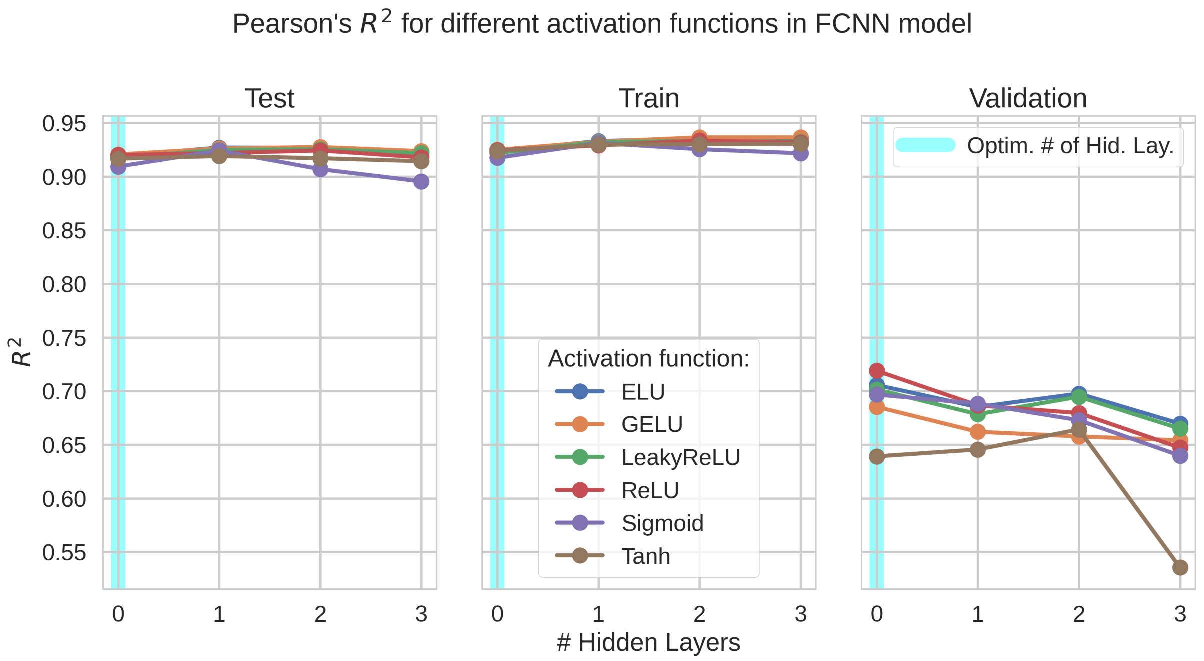

Figure S22: Comparison of the R^2 metrics on Train, Test and Validation sets for FCNN models with different activation functions and different number of hidden layers.

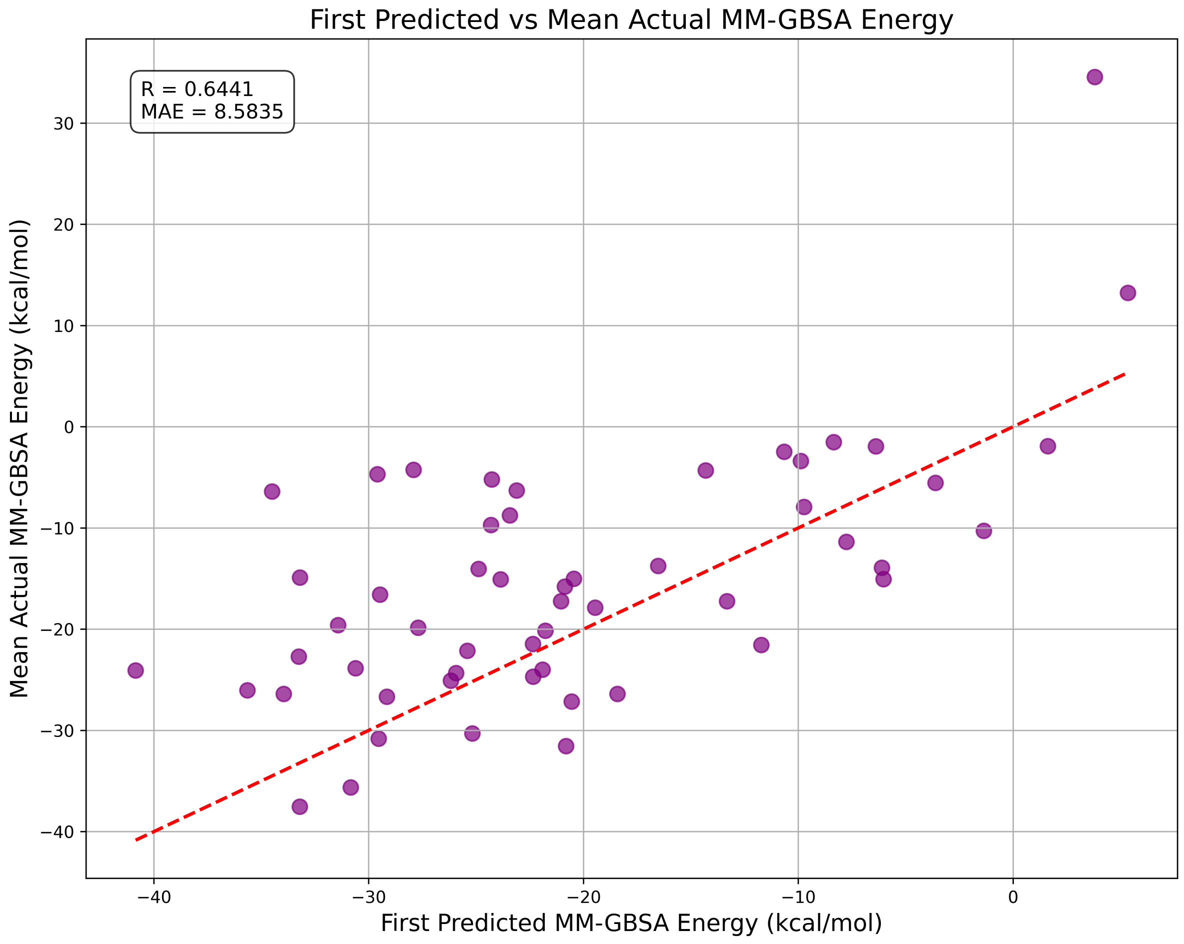

Figure S23: Comparison of predicted MM-GBSA binding free energies for first frames of MD simulation for cathepsin S-GAG complexes and actual mean MM-GBSA binding free energies for the same simulations for the FCNN model. The red dashed line indicates the ideal prediction (y = x).

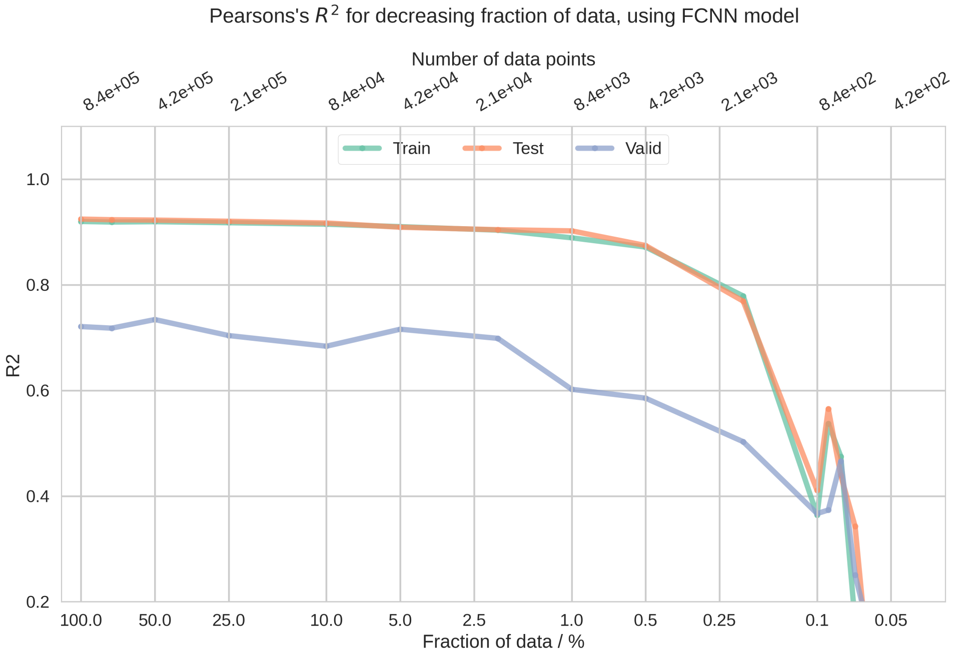

Figure S24: Comparison of the R^2 metrics on Train, Test and Validation sets for FCNN models with decreasing number of training data points.

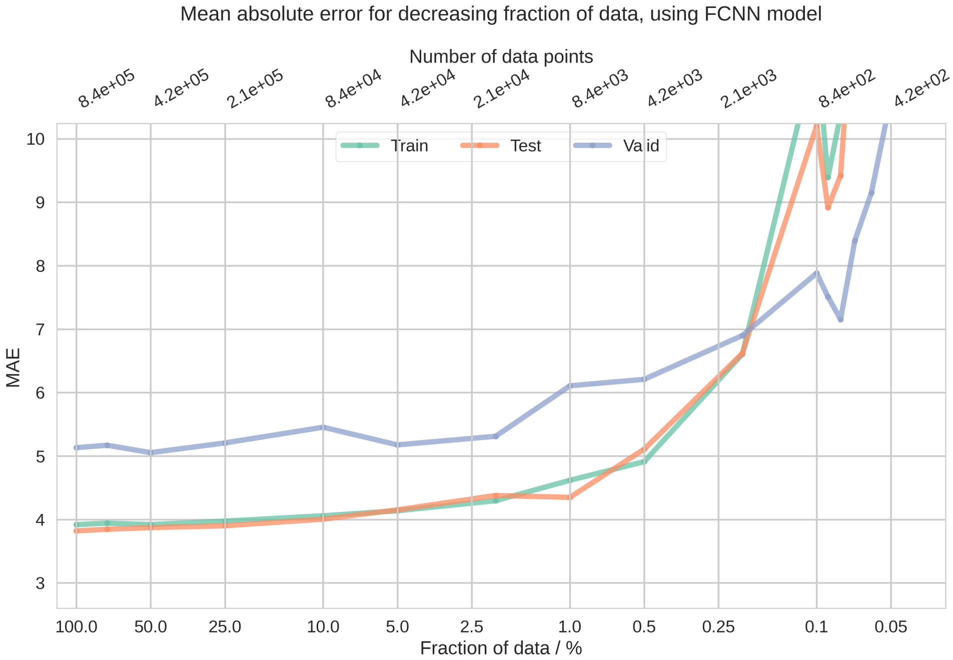

Figure S25: Comparison of the MAE in Train, Test and Validation sets for FCNN models with decreasing number of training data points.

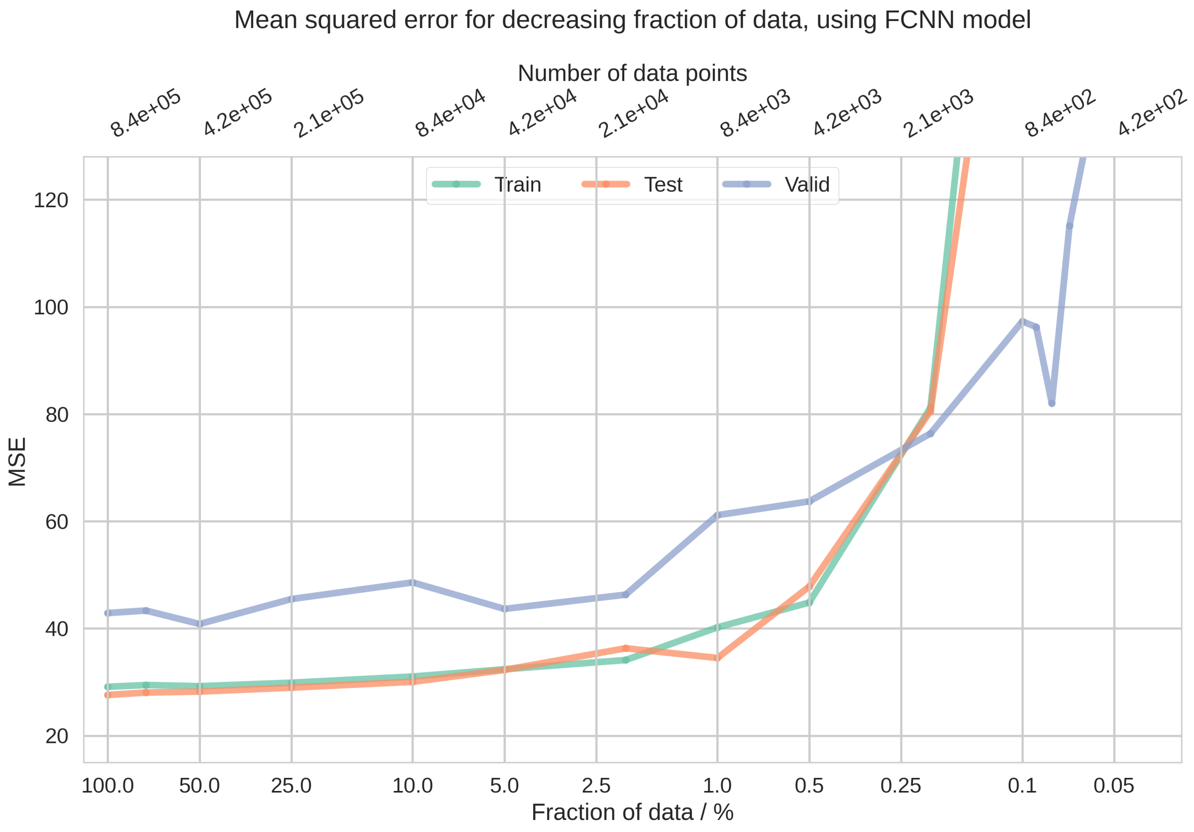

Figure S26: Comparison of the MSE in Train, Test and Validation sets for FCNN models with decreasing number of training data points.

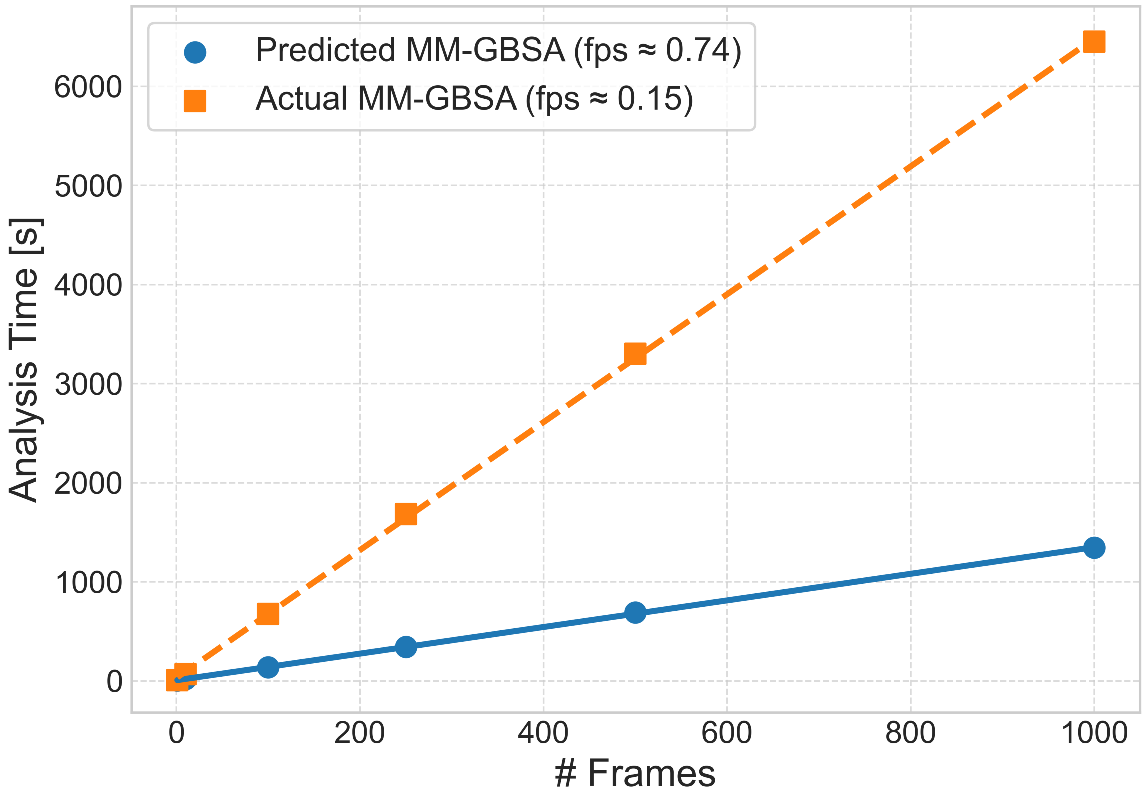

Figure S27: Scaling of analysis time with the number of frames for predicted MM-GBSA and conventional MM-GBSA calculations.

Table S1: Comparison of the R^2, MAE and MSE metrics for FCNN models with different dropout rates and different architectures trained to predict MM-GBSA energies for (pro)cathepsin-GAG complexes.

**Dropout rate**	**Number of hidden layers**	**Data Set**	$\mathbf{R^2}$	**MSE**	**MAE**
0.00	0	Train	0.9260	26.8672	3.8055
		Test	0.9269	26.7277	3.7976
		Validation	0.7041	45.4705	5.1740
	1	Train	0.9342	23.9095	3.6086
		Test	0.9351	23.7198	3.6027
		Validation	0.5684	66.3288	6.3258
	2	Train	0.9334	24.1686	3.6193
		Test	0.9342	24.0681	3.6159
		Validation	0.6406	55.2344	5.8934
	3	Train	0.9345	23.7693	3.5955
		Test	0.9353	23.6705	3.5933
		Validation	0.1294	133.8055	9.3252
0.01	0	Train	0.9200	29.1210	3.9184
		Test	0.9245	27.6044	3.8201
		Validation	0.7190	43.1918	5.1335
	1	Train	0.9219	28.3701	3.8853
		Test	0.9290	25.9467	3.7401
		Validation	0.6867	48.1464	5.5217
	2	Train	0.9243	27.5913	3.8420
		Test	0.9335	24.3277	3.6479
		Validation	0.6796	49.2473	5.5105
	3	Train	0.9179	29.9240	3.9303
		Test	0.9317	24.9615	3.6799
		Validation	0.6473	54.2056	5.8826
0.05	0	Train	0.9065	33.9314	4.2060
		Test	0.9161	30.6695	4.0127
		Validation	0.7113	44.3668	5.2814
	1	Train	0.9077	33.6349	4.2238
		Test	0.9247	27.5274	3.8397
		Validation	0.7086	44.7893	5.2781
	2	Train	0.8940	38.7017	4.4586
		Test	0.9215	28.6964	3.9173
		Validation	0.7002	46.0819	5.2895
	3	Train	0.8743	45.8007	4.7162
		Test	0.9227	28.2490	3.9245
		Validation	0.6863	48.2106	5.4345
0.30	0	Train	0.8660	48.9688	5.0299
		Test	0.9051	34.7072	4.2880
		Validation	0.7010	45.9520	5.3427
	1	Train	0.8157	67.1578	5.8129
		Test	0.8953	38.2786	4.4788
		Validation	0.6699	50.7369	5.6309
	2	Train	0.6756	117.9558	7.3574
		Test	0.8121	68.7132	5.5243
		Validation	0.6320	56.5583	5.8619
	3	Train	0.5833	151.0971	8.5406
		Test	0.7792	80.7375	6.2146
		Validation	0.5803	64.5027	6.3338

Table S2: Comparison of the R^2, MAE and MSE metrics for FCNN models with different activation functions and different architectures trained to predict MM-GBSA energies for (pro)cathepsin-GAG complexes.

**Activation function**	**Number of hidden layers**	**Data Set**	$\mathbf{R^2}$	**MSE**	**MAE**
ReLU	0	Train	0.9200	29.1210	3.9184
		Test	0.9245	27.6044	3.8201
		Validation	0.7190	43.1918	5.1335
	1	Train	0.9219	28.3701	3.8853
		Test	0.9290	25.9467	3.7401
		Validation	0.6867	48.1464	5.5217
	2	Train	0.9243	27.5913	3.8420
		Test	0.9335	24.3277	3.6479
		Validation	0.6796	49.2473	5.5105
	3	Train	0.9179	29.9240	3.9303
		Test	0.9317	24.9615	3.6799
		Validation	0.6473	54.2056	5.8826
ELU	0	Train	0.9190	29.4278	3.9475
		Test	0.9236	27.9336	3.8486
		Validation	0.7057	45.2297	5.3037
	1	Train	0.9270	26.5735	3.7770
		Test	0.9331	24.4492	3.6349
		Validation	0.6852	48.3843	5.4508
	2	Train	0.9267	26.6298	3.7880
		Test	0.9353	23.6423	3.6002
		Validation	0.6977	46.4572	5.3891
	3	Train	0.9234	27.9015	3.8461
		Test	0.9351	23.7143	3.5952
		Validation	0.6697	50.7692	5.5481
GELU	0	Train	0.9206	28.8292	3.9061
		Test	0.9249	27.4745	3.8210
		Validation	0.6854	48.3454	5.4896
	1	Train	0.9262	26.8163	3.7920
		Test	0.9324	24.7094	3.6669
		Validation	0.6621	51.9320	5.6802
	2	Train	0.9275	26.3782	3.7627
		Test	0.9365	23.2190	3.5657
		Validation	0.6578	52.5956	5.7235
	3	Train	0.9237	27.7262	3.8021
		Test	0.9365	23.2326	3.5666
		Validation	0.6541	53.1644	5.6575
⋮	⋮	⋮	⋮	⋮	⋮

Activation function	Number of hidden layers	Data Set	R^2	MSE	MAE
LeakyReLU	0	Train	0.9175	29.9667	3.9709
		Test	0.9220	28.5190	3.8874
		Validation	0.7015	45.8796	5.3299
	1	Train	0.9248	27.2817	3.8219
		Test	0.9320	24.8606	3.6820
		Validation	0.6785	49.4060	5.5149
	2	Train	0.9254	27.0839	3.7984
		Test	0.9331	24.4616	3.6409
		Validation	0.6948	46.9107	5.3606
	3	Train	0.9217	28.4325	3.8662
		Test	0.9324	24.7294	3.6609
		Validation	0.6651	51.4701	5.6687
Sigmoid	0	Train	0.9093	32.9671	4.2024
		Test	0.9175	30.1734	4.0065
		Validation	0.6968	46.5968	5.4131
	1	Train	0.9242	27.5131	3.8538
		Test	0.9313	25.1249	3.6997
		Validation	0.6884	47.8887	5.3358
	2	Train	0.9069	33.7967	4.1482
		Test	0.9255	27.2544	3.7536
		Validation	0.6729	50.2666	5.5092
	3	Train	0.8955	37.8268	4.2702
		Test	0.9217	28.6128	3.7931
		Validation	0.6399	55.3509	5.8835
Tanh	0	Train	0.9167	30.2936	4.0661
		Test	0.9238	27.8500	3.8560
		Validation	0.6392	56.8342	5.9981
	1	Train	0.9191	29.4103	4.0292
		Test	0.9298	25.6718	3.7207
		Validation	0.6456	54.4631	5.8655
	2	Train	0.9171	30.1508	4.0677
		Test	0.9302	25.5144	3.6967
		Validation	0.6643	51.6011	5.6613
	3	Train	0.9143	31.1735	4.1402
		Test	0.9305	25.4156	3.7046
		Validation	0.5356	71.3774	6.6243
